# Ru-Controlled Thymine Tautomerization Frozen by a k^1^(O)-, k^2^(N,O)-Metallacycle: An Experimental and Theoretical Approach

**DOI:** 10.3390/molecules28103983

**Published:** 2023-05-09

**Authors:** Silvia Bordoni, Riccardo Tarroni, Magda Monari, Stefano Cerini, Fabio Battaglia, Gabriele Micheletti, Carla Boga, Giacomo Drius

**Affiliations:** 1 Department of Industrial Chemistry ‘Toso Montanari’, Alma Mater Studiorum, Università di Bologna, Viale del Risorgimento 4, 40136 Bologna, Italy; riccardo.tarroni@unibo.it (R.T.); carla.boga@unibo.it (C.B.); giacomo.drius2@unibo.it (G.D.); 2 Health Sciences and Technologies Interdepartmental Center for Industrial Research (CIRI SDV), University of Bologna, 40126 Bologna, Italy; 3 Department of Chemistry ‘Giacomo Ciamician’, Alma Mater Studiorum, Università di Bologna, Via Selmi 2, 40126 Bologna, Italy; magda.monari@unibo.it

**Keywords:** ruthenium, Ru(II), thymine, (N,O) chelation, self-assembly, X-ray crystal structure, DFT, H-bonding network

## Abstract

The reaction of *mer*-(Ru(H)_2_(CO)(PPh_3_)_3_) (**1**) with one equivalent of thymine acetic acid (THAcH) unexpectedly produces the macrocyclic dimer k^1^(O), k^2^(N,O)-(Ru(CO)(PPh_3_)_2_THAc)_2_ (**4**) and, concomitantly, the doubly coordinated species k^1^(O), k^2^(O,O)-(Ru(CO)(PPh_3_)_2_THAc) (**5**). The reaction promptly forms a complicated mixture of Ru-coordinated mononuclear species. With the aim of shedding some light in this context, two plausible reaction paths were proposed by attributing the isolated or spectroscopically intercepted intermediates on the basis of DFT-calculated energetic considerations. The cleavage of the sterically demanding equatorial phosphine in the *mer*-species releases enough energy to enable self-aggregation, producing the stable, symmetric 14-membered binuclear macrocycle of **4**. The k^1^-acetate iminol (C=N-OH) unit of the *mer*-tautomer k^1^(O)-(Ru(CO)(PPh_3_)_2_(THAc)) (**2**) likely exhibits a stronger nucleophilic aptitude than the prevalent N(H)-C(O) amido species, thus accomplishing extra stabilization through concomitant k^2^(N,O)-thymine heteroleptic side-chelation. Furthermore, both the ESI-Ms and IR simulation spectra validated the related dimeric arrangement in solution, in agreement with the X-ray determination of the structure. The latter showed tautomerization to the iminol form. The ^1^H NMR spectra in chlorinated solvents of the kinetic mixture showed the simultaneous presence of **4** and the doubly coordinated **5**, in rather similar amounts. THAcH added in excess preferentially reacts with **2** or *trans*-k^2^(O,O)-(RuH(CO)(PPh_3_)_2_THAc) (**3**) rather than attacking the starting Complex **1**, promptly forming the species of **5**. The proposed reaction paths were inferred by spectroscopically monitoring the intermediate species, for which the results were strongly dependent on the of conditions the reaction (stoichiometry, solvent polarity, time, and the concentration of the mixture). The selected mechanism proved to be more reliable, due to the final dimeric product stereochemistry.

## 1. Introduction

**Figure 1:**
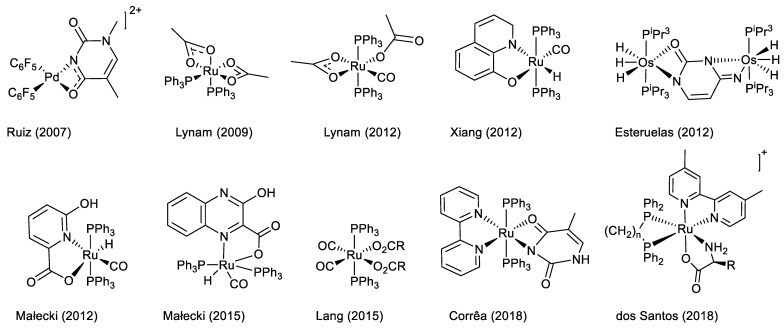
Selected examples of the previously reported homoleptic k^2^-(O,O) and heteroleptic k^2^-(N,O)-cyclometalated heterocyclic complexes. Adapted with permission from Refs. [4,5,6,7,8,10,11,13,15]. 2015 Małecki J.G., 2012 Małecki J.G., 2012 Xiang J., 2007 Ruiz J., 2012 Esteruelas M.A., 2018 Corrêa R.S., 2018 dos Santos E.R., 2009 Lynam, J.M., 2012 Lynam J.M., 2015 Lang, H.

We recently reported the synthesis, crystal structure, and theoretical investigations of the reaction between the dihydride complex (Ru(H)_2_(CO)(PPh_3_)_3_) (**1**) and two equivalents of thymine acetic acid (THAcH) that promptly produced k^1^(O), k^2^(O,O)-(Ru(CO)(PPh_3_)_2(_THAc)_2_) (**5**) with a good yield (90%), simultaneously bearing monohapto- and dihapto-acetate with vicinal phosphine ligands [1]. Analogous cyclo-metalated (N,O)- [2,3,4,5,6,7,8,9,10,11] and (O,O)- [12,13,14] chelated species have been previously reported by other groups. (Figure 1).

These compounds have claimed particular interest, particularly concerning their catalytic features such as the isomerization or reduction of propargylic alcohols [13,14,15] and transfer hydrogenation [16]. Moreover, in the last 10 years, they have demonstrated remarkable bioactivity as anticancer agents [9,11], ascribable to the exhibited versatility and flexibility (mainly due to the facile cleavage of phosphine ligands and their relocation), but also to their thermodynamic robustness as carriers, due to the function of chelated carboxyl. Recently, we also described the pivotal role of Ru’s coordination in the derivatives of thymine acetate (THAc), even in the absence of electron conjugation, due to the N-methylene spacer between thymine ring and the tethered acetate [1]. The role of metal coordination and the H-binding interactions network both result in affecting the transfer of H by triggering the transformation from the prevalent (C(O)NH) keto-amino to the rare tautomeric (N=C(OH)) iminol fragment. The iminol/amido isomer ratio has been evaluated to be 33% through the presence of low-shifted ^1^H NMR signals in the interval of 12.0–7.7 ppm, as observed in (k^1^-**2a,b** + k^2^-**3**) numerous mixtures of various preparative paths and attributed to the iminol forms stabilized by intra- or inter-H-binding network. Analogous results have been accomplished under the same conditions by doubling the concentration of the reaction mixture, albeit with a remarkably reduced yield (25%).

## 2. Results and Discussion

### 2.1. Synthesis of the Complexes cis_P,P_-k^1^(O), k^2^(N,O)-(Ru(CO)(PPh_3_)_2_(THAc)_2_) (***5***) + cis_P,P_- k^1^(O), k^2^(N,O)-(Ru(CO)(PPh_3_)_2_THAc)_2_ (***4***)

Through the addition of one equivalent of THAcH to (Ru(H)_2_(CO)(PPh_3_)_3_) (**1**) [17] in refluxing toluene, the solution gradually darkened from the initial light brown, with a slight concomitant release of gas. According to IR monitoring, after 3 h, the reaction had completed, due to the disappearance of the reactants. Upon the removal of the solvent and a work-up by filtration on a celite pad, a mixture of the known k^1^(O),k^2^(O,O)-(Ru(CO)(PPh_3_)_2_(THAc)_2_) (**5**) and the novel dimeric species *cis*_P,P_-,k^1^(O),k^2^(N,O)-(Ru(CO)(PPh_3_)_2_(THAc))_2_ (**4**) were detected by the ^1^H NMR spectra. After standing at −20 °C for 30 days, the CDCl_3_ solution produced crystals of **4** (33%) and, concomitantly, **5** (18%), both exhibiting an unexpected *cis*-conformation for the phosphine ligands in the crystalline structures.

**Figure 2:**
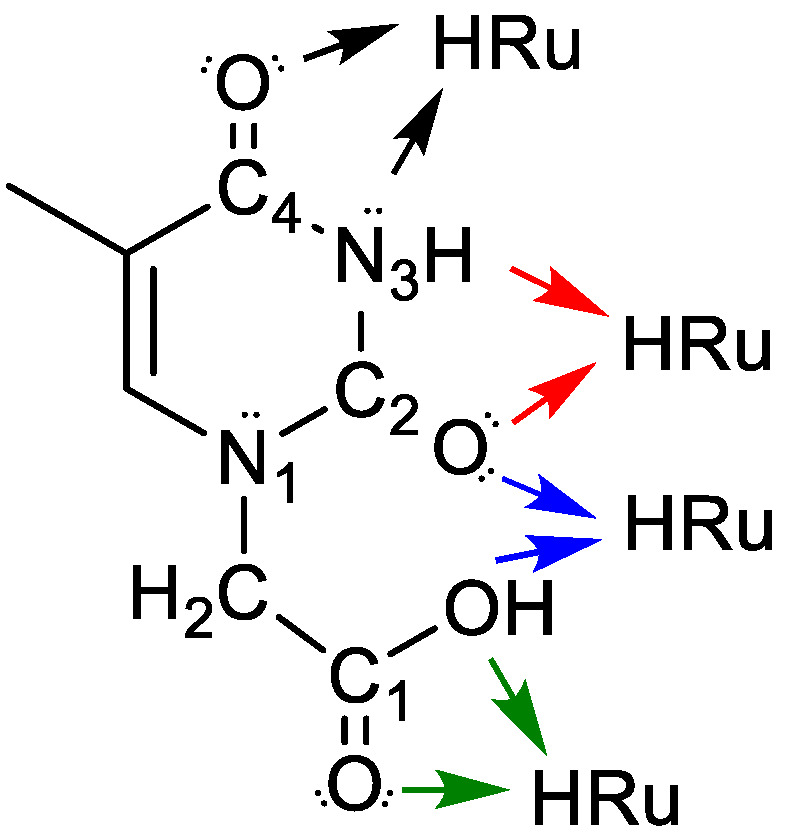
Thymine acetic acid attacks metal complexes via chelation, producing four distinct four-membered metallacycles: two of them show (N,O)-heteroleptic coordination (those designated by red and black arrows involve N3,O4 and N3,O2, respectively), while one (green) concerns the homoleptic k^2^-(O,O). Analogous chelation through (O,O2) (blue arrows) involves the possible formation of two distinct metal heptacycles.

As illustrated in Figure 2, the THAcH molecule shows, in theory, four different chelate-coordinative modes (indicated by the different colored arrows in Figure 2). Each of them potentially could generate a pair of diastereoisomers, depending on the reciprocal opposite positions of the H/CO ligand.

Indeed, every dihapto-coordinative mode generates two distinct metallacycles, except for the k^2^(O,O)-heptacycle (blue arrows) or the k^2^(O,O)-carboxy-chelate derivatives (green).

Due to the strong acidity of the carboxy moiety, the first attack of the thymine derivatives is directed toward the Ru-H moiety, achieving the monohapto-acetate complexes k^1^(O)-(RuH(CO)(PPh_3_)_3_THAc) (**2a,b**) attributed to the typical high-shifted residual hydride signals in the δ −4/−8 interval.

**Figure 3:**
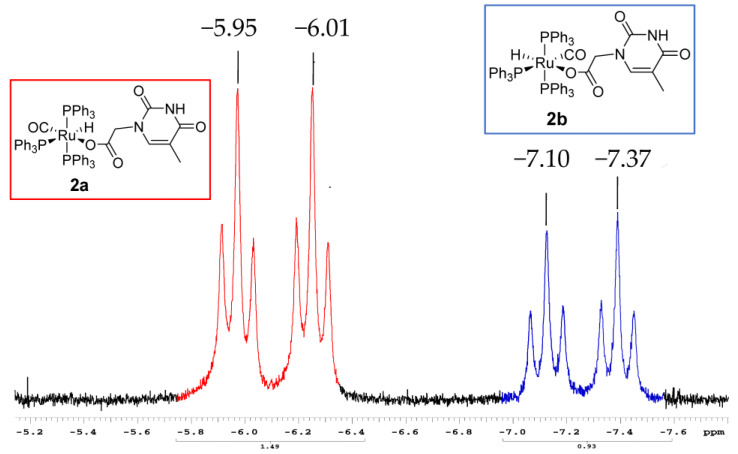
The newly formed monohapto-acetate intermediates **2a** (red) and **2b** (blue) are supported by the two distinct 1:0.8 ^1^H NMR multiplet signals centered at δ = −6.1 and −7.2 in the hydride region with ^2^J_H-P*trans*_ = 111 Hz and ^2^J_H-P*cis*_ = 24 Hz.

The observed ^1^H NMR doublets of the triplets have been assigned to the monohapto-diastereoisomers **2a** and **2b**, depending on the opposite position of the H/CO (Figure 3). A push–pull electron density motif enabled us to distinguish the more stable **2a**, which shows CO *trans*- to the donor acetate moiety from **2b.** The ratio between the two diastereoisomers depends on the solvent’s polarity, which is able to induce **2** to further reactivity. For example, upon standing at room temperature the mixture of **2a,b** in a CDCl_3_ solution, within the more deshielded signal in the range of −(5.8/6.4) ppm related to the isomer **2b** was more intense than that of **2a**, indicating the faster reactivity of the latter. In this context, the monohapto species spontaneously evolved at room temperature in 90–120 min into the chelate derivative **3**, as shown by the remarkable Ru-H shift of the ^1^H NMR triplet, which was greatly influenced by the shielding due to the dihapto coordination. The partially overlapped triplets, observed in the δ = −(19/16) interval, are hydride units typically coupled with the mutually located and magnetically equivalent phosphine ligands at an axial position (^2^J_HP_ ≅ 24 Hz).

**Figure 4:**
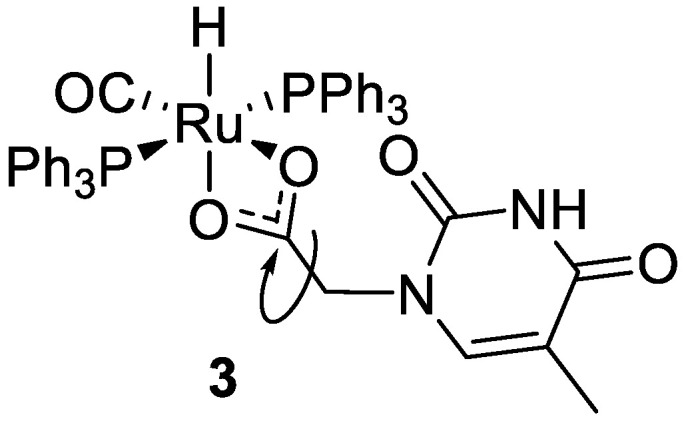
The rotamers relative to the isomers of **3** due to the low energy torsion barrier, which was calculated to be 10 kJ mol^−1^.

Under the general assumption that more stable species correspond to the major ^1^H NMR hydride signals, the very broad major triplet at −17.01 ppm (in green) was assigned to the entropically favorable complex **3**. The latter revealed uncountable conformations due to the mobility of the k^2^(O,O)-side arm, with low energy rotation (10 kJ mol^−1^) around the C-C bond between the methylene spacer and the carboxy unit (Figure 4). Both experimental and theoretical studies of the rearrangement have been detailed in our recent publication [1].

**Figure 5:**
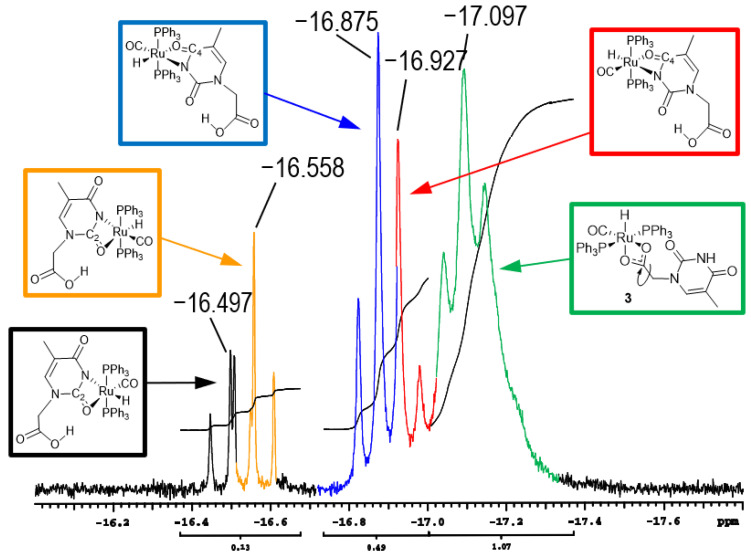
The ^1^H NMR spectrum in the hydride region. The large triplet depicted in green (δ = −17.10) is attributed to the k^2^(O,O)-**3**, while the adjacent red (δ = −16.93) and blue (δ = −16.87) boxes are related to the heteroleptic **6b** and the minor signal analogous to **6a**.The adjacent overlapped triplets are assigned to the heteroleptic chelated k^2^(N,O) species (orange, **7a**, δ = −16.56; black, **7b**, δ = −16.50 ppm). The newly formed four-membered metallacycles are generated by the action of heteroleptic chelation, depending on the involvement of CO(4) or CO(2). These species may be further stabilized by the C(O)OH−−−(O)C-Ru intramolecular H interaction between the carboxy group and the function of the ketone ring as suggested by the low-shifted weak but sharp signal at δ = 11.2, tentatively attributed to H-bond interactions [1].

However, in our case, the reaction monitored for 60 min in refluxing toluene rapidly evolved into a complicated kinetic mixture (see Figure 5), the nature of which was revealed by attributing the ^1^H NMR signals on the basis of the energetic considerations obtained by means of the theoretical DFT calculations.

**Scheme 1:**
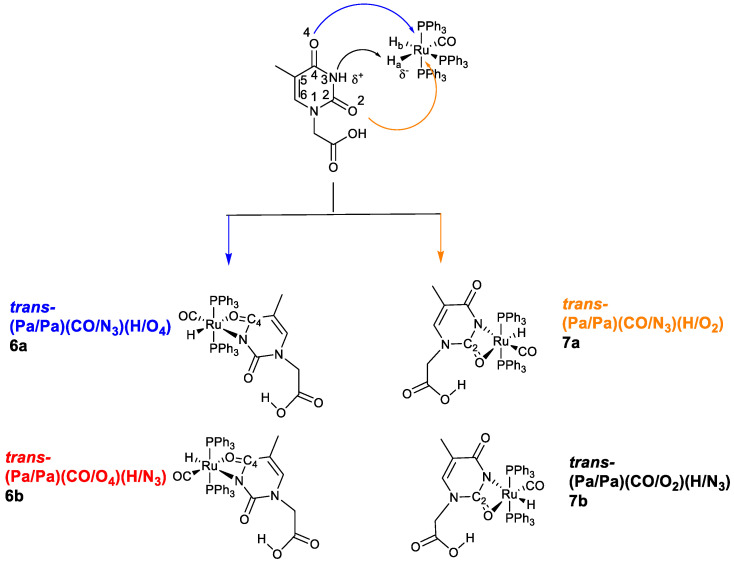
The major triplet sets at δ = −16.87 (blue) and −16.56 (orange) belong to *k*^2^(N3,O4)-(RuH(CO)(PPh_3_)_2_THAc) (**6**), giving rise to distinct diastereoisomers depending on the location of H/CO in **6a**, **6b**; the carbonyl opposite to the donor N atom was less energetic and more stable (Figure 5). The minor triplet at δ = −16.93 (red) partly overlapping with an analogous signal centered at δ = −16.50 (black) is attributed to the isomers **7a** and **7b** (23.9 and 24.5 kJ mol^−1^, respectively). The heteronuclear k^2^(N,O) complexes have been named as described in the theoretical section (see Figure S11).

**Figure 6:**
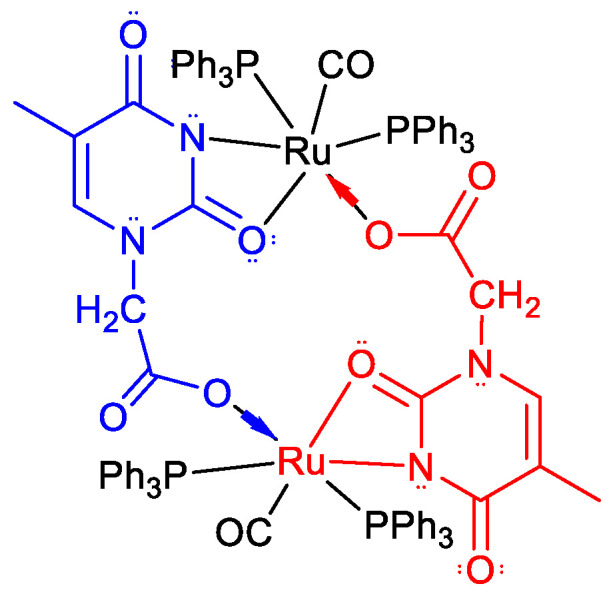
As a proposed alternative path to produce the macrocycle of **4**, the initially formed k^2^(N,O) metal fragments, designated by opposite stereogenic Ru centers, might mutually self-aggregate.

Depending on the intermediates formed by the chelation of the thymine ring, *k*^2^(N3,O4)-**6** or *k*^2^(N3,O2)-**7**, a further possible course of the reaction may be postulated (Scheme 1). However, in our case, these intermediates were fairly elusive, as suggested by the relative energy factors calculated by DFT in the case of *trans*- (**6b**: 22.1, **7a**: 23.9, **7b**: 24.5, **6a**: 28.8 kJ mol^−1^) and *cis*-stereogeometry. Further, the opposite stereogenic center needs to be selected to explain the resulting geometry observed in the dimer **4** (Figure 6).

The (N,O)-chelated species are formed by the acid–base reaction between the N(3)H unit with the hydridic H-Ru fragment with the less congested thymine CO(4), which displaces the vicinal phosphine ligand in the *mer*-species. The resulting complexes commonly show *trans_PP_*-stereochemistry. The more stable configurations are likely formed when the donor N-atom is *trans* located to the Ru-CO acceptor ligand (**6a**). The minor signals can instead be attributed to the diastereoisomers *k*^2^(N3,O2)-(RuH(CO)(PPh_3_)_2_THAc) (**7**).

**Scheme 2:**
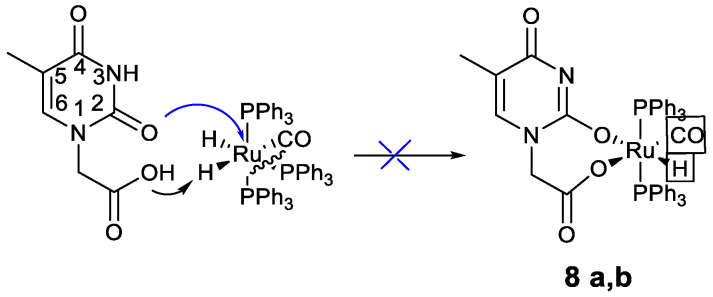
The seven-membered k^2^(O,O2)-**8** species that were never observed. The inversion between the adjacent H and CO ligands would produce distinct isomers with relatively high energy (26.3 (**8a**) and 39.0 (**8b**) kJ mol^−1^).

In the latter case, closure of the ring is not favorable for steric congestion, taking into account that the thymine O(2) atom is partially engaged in the H-bond with the carboxyl- nit. In this context, the strained seven-membered species **8** (Scheme 2), which is probably derived from the chelation of k^2^(O,O)-, was never observed, in agreement with the high calculated energy (39.0 and 26.3 kJ mol^−1^).

The nature of the THAc–coordinated species k^2^(O,O)-**3** was supported by the ESI-Ms analysis in MeOH. The occurrence of the cationic stable species (Ru(CO)(PPh_3_)_2_(k^2^-THAc))^+^, where (M − H)^+^ = 837 m/z, and fragments generated by the loss of the THAc (*m*/*z* = 653) and CO (*m*/*z* = 625) ligands, respectively, are also observable (Supplemental Figures S9 and S10). Signals related to the rapid oxidation of PPh_3_ under the experimental conditions were also revealed (*m*/*z* = 301 and 279, OPPh_3_). The loss of a hydride to give a (M − H)^+^ peak is common for this kind of complex, as already been reported [6].

The spectrum of the less stable monohapto species instead shows the occurrence of the cationic fragment (*m*/*z* = 1123) relative to (M + Na)^+^ (Supplemental Figure S8).

The names (colors) for each diastereoisomer imply the stereo-location of the reciprocal axial phosphine (Pa); the chelated heteroatoms are labeled with the same numbers as shown in Figure 2, and the final atoms in parentheses separated by the hyphen indicate the mutual *trans*-position. Through self-coupling, the orange or blue major isomers of *trans*-**6a**, **7a** could also generate the macrocycle of **4** (Figure 6). However, the higher energies and geometric criteria required to select the opposite stereogenic centers to be coupled make the formation of the dimer through this path significantly unfavorable.

### 2.2. Characterization of **4** and **5**

#### 2.2.1. ESI-Ms of **4**

**Figure 7:**
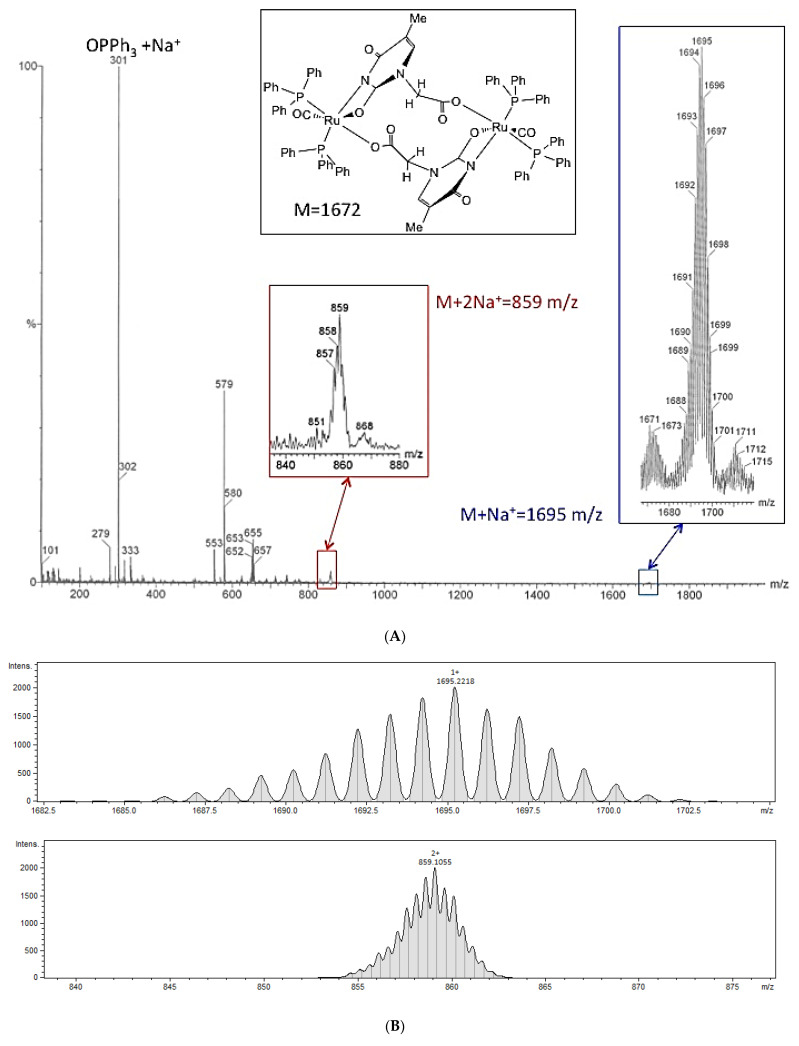
(**A**) The ESI-Ms spectra (positive mode) support the formation of a dimeric form, stabilized by the ring compenetrating the k^1^(O)-**2** metal fragments, as observed by the signal at 859 (M + 2Na)^2+^. (**B**) Simulation of ESI-Ms (positive mode) relative to **4**.

The cationic ESI mass spectrum of the dimer of **4** presented a pattern motif analogous to the parent k^1^-fragment, but was significantly broader and unresolved, typical of species generated from symmetrically fragmented dimeric molecules (see Figure 7A).

#### 2.2.2. Description of the X-Ray Crystal Structure of **4**

**Figure 8:**
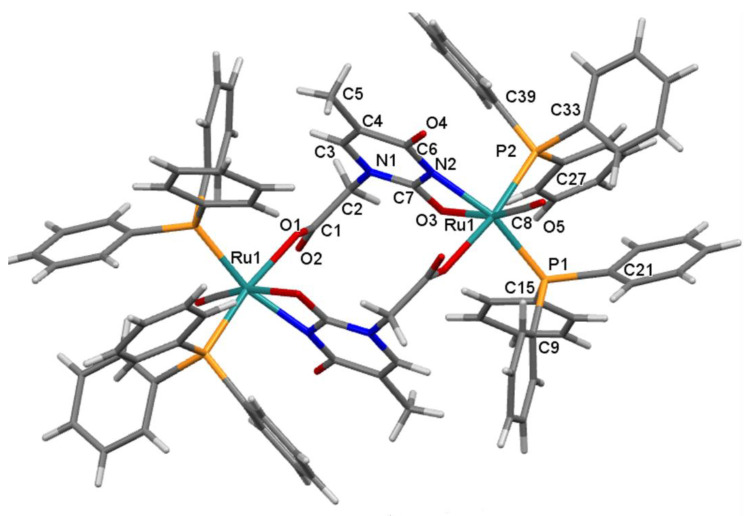
X-ray crystal structure of k^2^(N,O),k^1^(O)-(Ru(CO)(PPh_3_)_2_THAc)_2_ (**4**). The crystallographic inversion center is located midway between the Ru atoms.

The X-ray molecular structure of **4** is shown in Figure 8, and the related bond lengths and angles are reported in Table 1. The molecule sits around an inversion center located midway between the Ru atoms, and consists of two Ru(PPh_3_)_2_(CO) units bridged by two thymine acetate groups. The geometry of the coordination at the Ru centers is a distorted octahedron with the PPh_3_ ligands adopting an unusual *cis*-configuration, one CO, and two bridging thymine acetate groups. The thymine acetate units are arranged in an antiparallel mode, coordinated to each Ru atom through one oxygen of the carboxylate moiety of one ligand and chelated by the (N,O) atoms of the thymine belonging to the second bridging ligand, thus forming a 14-membered dimetallacycle.

For the thymine acetate, this bridging mode is unprecedented, since the most common bridging mode is that involving only carboxylate oxygens, as has been found, for example, in a series of copper complexes (Cu_2_(μ-OOCCH_2_-T)_4_(G)_2_) (T = thymine-1-acetate, G = dimethylformamide, etc.) [19]. As a consequence of the bidentate coordination of O3 and N2 at the ruthenium atom, a lengthening of the C7-O3 bond (C7-O3 1.280(8) Å) associated with a shortening of the C7-N2 bond (C7-N2 1.341(9) Å) was observed in comparison with the corresponding bond distances in the free THAcH [18].

**Figure 9:**
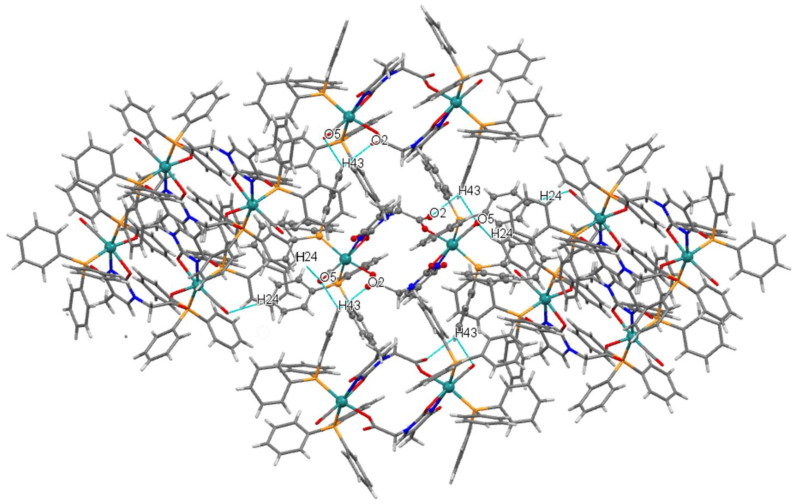
View of the environment of **4** (in the center, ball and stick model) showing the intermolecular C-H...O hydrogen bonds (light blue dotted lines) involving the central molecule and the six neighboring molecules.

Furthermore **4** established several nonclassical intermolecular H bonds (Figure 9) involving the uncoordinated carboxylic O atoms of the thymine acetates with the aromatic hydrogens (O2…H43-C43) and the O of the carbonyl group bound to Ru that interacted simultaneously with the aromatic hydrogens H43 and H24 (O5…H43-C43 and O5…H4-C24), with each molecule engaging with six neighboring H bonds. The supramolecular network is controlled by intermolecular H bonds, as shown by the packing of the crystals (Figure S14).

### 2.3. Proposed Reaction Path

**Scheme 3:**
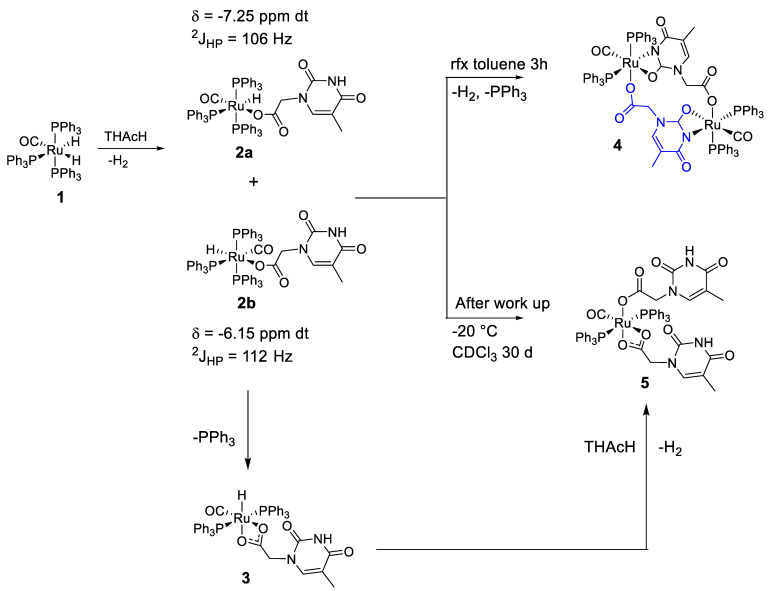
Scheme of the distinct courses of the reaction to explain the concomitant formation of the mixture composed of the dimer k^2^(N,O), k^1^(O)−(Ru(CO)(PPh_3_)_2_THAc)_2_ (**4**) and the doubly coordinated k^1^(O), k^2^(O,O)−(Ru(CO)(PPh_3_)_2_(THAc)_2_) (**5**).

Herein, we describe the reactivity of the dihydride (Ru(H)_2_(CO)(PPh_3_)_3_) complex, **1,** in refluxing toluene with an equimolar amount of THAcH (Scheme 3), unexpectedly producing the dinuclear 14-membered symmetric macrocycle of the type k^2^(N,O), k^1^(O)-THAc-(Ru(CO)(PPh_3_)_2_)_2_ (**4**).

The course of the reaction, likely driven by the reciprocal association of the *mer*-k^1^(O)-monohapto species of **2**, is induced by:

- The rapid release of H_2_ from the reaction of the acidic NH-thymine ring and the Ru-H fragment;
- The dissociation of a PPh_3_ ligand through the action of the N-nucleophilic addition to the center of the Ru;
- Stereo-rearrangement of the phosphine ligands to achieve the less encumbered *cis_P,P_* conformation, which was ultimately exhibited by the Ru skeleton in the solid state.

The cleavage of the vicinal phosphine in the *mer*-k^1^(O)-**2** complex to form the chelate *trans*-k^2^(O,O)-(RuH(CO)(PPh_3_)_2_THAc), **3**, is, however, a competitive route favoring entropy. In solution, the phosphine ligands occupy a mutual *trans* position, as confirmed by the relative triplet in the ^1^H NMR spectra. Thus, the *trans*–*cis* rearrangement of phosphine is required to explain the stereochemistry of the crystallized macromolecule of **4**. The precursor mixture, after standing in a CDCl_3_ solution in an NMR tube for 30 days at −20 °C, formed crystals of the dimeric species of **4** together with a minor amount of the monohapto-dihapto species **5**. This result suggested that the k^2^-chelated species of **3** favoring entropy exhibited faster incorporation of a further THAcH molecule into the Ru skeleton, promoted by the stronger hydridic character than the parent, **1**. Thus, the formation of the unexpected dimetallacycle k^1^(O), k^2^(N,O)-**4** and the monomeric species k^1^(O), k^2^(O,O)-**5** can be attributed to the influence of distinctive features:

- The poor solubility of the large molecule of **4** in chlorinated polar solvents, such as CH_2_Cl_2_, or CDCl_3_;
- The stronger nucleophilic ability to promote self-coupling, shown by the tautomeric iminol species, compared with the stable keto-amino form.

On the other hand, the enhanced hydridic character (δ = −16.5) of the chelate *trans_P,P_,* k^2^(O,O)-(RuH(CO) (PPh_3_)_2_THAc) (**3**), due to the influence of the dihapto-acetate ligand, and the minimized inter-ligand encumbrance, in agreement with the previous theoretical calculations for the energy of torsion (10 kJ mol^−1^) [1], are factors which promote both the extra stabilization of **4** and the competitive formation of **5.** The resulting *cis*_P,P_ rearrangement, which was exhibited by the crowded species of **5,** was also observed also in the self-aggregated multihapto complex, **4**.

#### 2.3.1. IR Spectra

**Figure 10:**
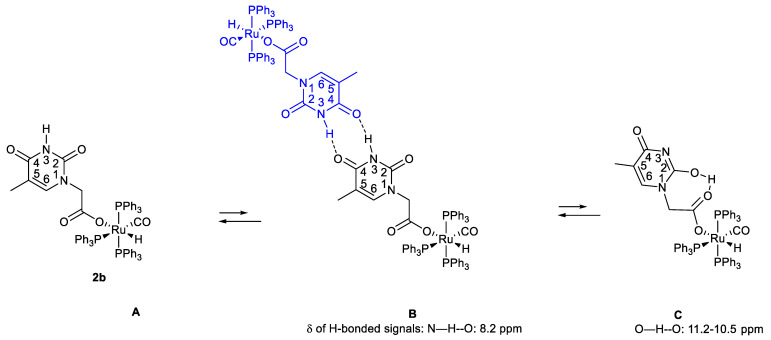
Typical intra- or intermolecular H–bonds in the monohapto k^1^(O)–**2** precursor [1]. (**A**) neither intra– nor intermolecular H–bond is present. (**B**) exclusively intermolecular H–bonds of Watson–Crick type are present in the reciprocal interactions between the species. (**C**) intramolecular H–bond between monohapto carboxyl unit and ring CO_2_ carbonyl function is evidenced.

In the THAcH ring, the CO(2) and CO(4) groups may act as hydrogen acceptors, whereas the amino N3 is a hydrogen donor. Due to the forms of isoenergetic resonance, it is commonly assumed that the lactim–lactam tautomerism prevails over the enol–keto equilibrium. In a previous report, the absorption at 1918 cm^−1^ was attributed to a mixture of **2**, while more extensive degradation was exclusively assigned to **2a** in keto form (see Supplemental Figure S6). The IR spectrum of **2a**, indicated as a precursor of the metallacycle **4**, shows a pattern with 1683 (CO asym), 1654 (CO sym), and 1362 (C-O) cm^−1^ bands, typical of the structure of monohapto-acetate. The Ru-carbonyl moiety appeared at 1918 cm^−1^ with weak broad absorption of Ru-H at ν = 2044. The acidic nature of the lactam form increased in the tautomerized lactim form through the presence of the iminol-(HO-C=N) moiety. Likewise, the imino-nucleophilic aptitude, permitting self-aggregation towards the center of Ru’s center, was strongly enhanced. We believe that the tautomeric equilibrium from the lactam to the lactim forms was triggered by both the intramolecular interaction of H of the O-H-O type and by the extensive intermolecular H-binding network. The key to the rearrangement of the Ru-monohapto-thymine acetate **2a** to the minor tautomeric iminol species relies on the promotion of H-transfer to the stable lactim form. On the other hand, the growing IR absorbance at ν = 1511 (w) in the k^1^-acetate precursor **2b** may be associated with the Ru-CO at ν = 1930 and the weak carbonyl thymine band observed at 1737 cm^−1^, indicating the tautomerized transformation towards the “transient” lactim –((HO)C=N-C(O)NH) species (see Supplemental Figure S4). It is conceivable that the N-imine moiety of **2b** may exhibit a nucleophilic aptitude to the center of Ru which is stronger than that of the stable keto-amino species, thus favoring a concerted cleavage of the opposite equatorial phosphine to alleviate steric congestion. This process is triggered by multiple intermolecular interactions, which shuttle the transfer of H to promote the transformation to the iminol forms [20,21,22,23,24,25,26,27,28,29]. The coordination of Ru enables the stabilization of the unstable tautomer through the intramolecular interaction o fH (O-H–––(O)C-ORu) between the tethered carboxy unit and the C2(OH) iminol moiety of **C** (Figure 10), as suggested by the observed high-shifted signal at δ = 11.2. The intermolecularly bound keto forms of **B**, which appeared at δ = 8.2, is the typical NH—(O)C Watson–Crick type bond [1].

As a clue to its potential anticancer activity, the formation of minor iminol–tautomers may induce mismatching of the pairs of DNA nucleobases. The process of tautomerization is commonly recognized to induce remarkable consequences for the structure of DNA [30,31,32,33,34,35,36,37,38,39,40,41,42,43,44]. Since the canonical keto-amino isomers define the structure of the H–bond for Watson–Crick nucleobase pairing, the minor tautomeric species, when impaired during replication, may lead to mutagenesis [45,46,47,48]. Plausibly, both the intra– or inter–H–bonding networks of the Ru-coordinated k^1^(O)-THAc may be responsible for enol–thymine tautomerization, which ultimately produces the kinetically driven self-assembled Ru macrocycle. Further, the entire structure exhibits extra stabilization through the mutual coordination of the heteroleptic thymine ring k^2^(N3,O2). We suppose that, as a biochemical implication, the rare tautomeric forms are likely to act in the replication of mismatched DNA nucleobases. Unfortunately, the great insolubility precludes any attempts at biochemical tests. Further applications might be in trapping small molecules such as solvents (CHCl_3_, cyclohexane, or EtOH), as observed by X–rays of the crystallographic cells in different preparations, or by sequestering CO_2_ or alkaline metals such as Na^+^ or K^+^, which may be confined in the 14-membered large metallacycle. The central cavity, where the distance between the carboxyl C atoms is 557 pm, exhibited similar dimensions to zeolite holes, as revealed by the space-filling molecular model (Supplemental Figure S12). However, the sterically demanding phosphine ligands can regulate the entrances by closing the cyclo–metalated ring, and this process may be dynamic, owing to the flexible motion of the related P-phenyl rings.

**Figure 11:**
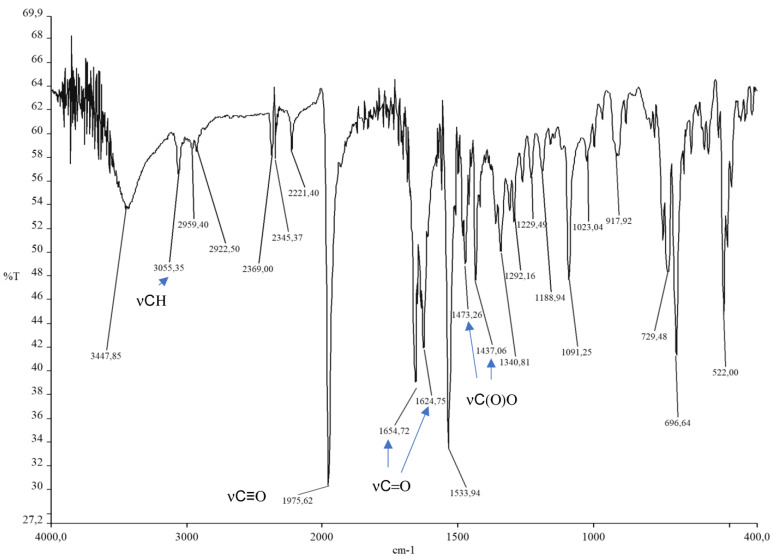
IR spectrum of the macrocycle dimer of **4**. The sharp IR band at 1534 cm^–1^ indicates the presence of the iminol form, which is attributable to the O-C=N IR stretching.

The IR pattern of acetate units, which reciprocally penetrate the opposite centers of Ru, were influenced by the four-membered dihapto–heteroleptic k^2^(N,O)–chelate (**6** and **7**), since the electrons’ density was strongly affected by the N-donor atom, revealing distinctive bands at 1654 and 1624 cm^−1^ assigned to the asymmetrical and symmetrical stretching modes of carboxy, respectively, with ν = 1340 cm^−1^ for the ν of C-O. The intense sharp band at ν = 1975 cm^−1^ of dimer **4** (Figure 11) was indicative of the presence of Ru-CO. Furthermore, the bending absorption at 1091 cm^−1^ likely generated an overtone at 2222 cm^−1^, the attribution of which was suggested by the IR simulation calculated by DFT (see Supplemental Figure S13), which distinguished the keto form of absorption from the tautomeric form of enol, confirming the occurrence of the latter.

#### 2.3.2. NMR Characterization

As revealed by the Ru-H triplet at −16.9 ppm, the remaining solution, composed of the related isomer, **2b**, underwent thermodynamically driven evolution of the chelate k^2^(O,O)-**3**. Meanwhile the insoluble microcrystalline white powder of **4** was dissolved in a 1:1 EtOAc–CH_2_Cl_2_ solvent mixture and purified upon filtration on a celite pad. The resulting compound showed ^1^H NMR signals at δ 5.75, 3.20, and 1.91 (see Supplemental Figure S1) in CDCl_3_, attributed, respectively, to the methyne, N-methylene, and methyl groups. Due to the extreme insolubility, any attempts to dissolve the microcrystalline white powder in deuterated Me_2_SO (dielectric constant ε = 46.7) or MeOH (dielectric constant ε = 32.6) led exclusively to rapid decomposition within 36 h at room temperature. However, the ^31^P NMR spectrum of CDCl_3_ revealed two residual doublets at 44.1 and 47.6 ppm assigned to the *cis*-located phosphine ligands of **4**, and a broad signal at 38.4 ppm attributable to the decomposition to the chelate precursor, *trans*-k^2^(O,O)-((CO)(PPh_3_)_2_(THAc)), which was around δ = 29 (see Supplemental Figure S2) was the predominant signal, due to the OPPh_3_-oxidized phosphine released from the *mer*-k^1^(O)-**2a**.

#### 2.3.3. DFT Calculations

**Scheme 4:**
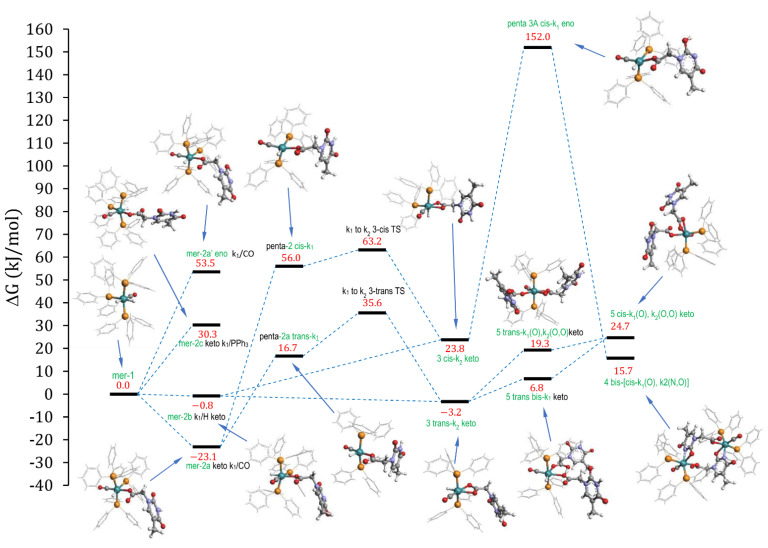
DFT calculations of the intercepted (*trans*−forms) or guessed species (*cis*−forms) for the mechanism sketched in Scheme 3, with plausible transition states and intermediates leading to **4** and **5**.

To shed some light on the unexpected results and the relative stereochemistry, further theoretical calculations were run by adopting a more accurate density-functional approach and by introducing the effects of the solvent and the temperature to mimic the actual transformation (see Scheme 4).

**Figure 12:**
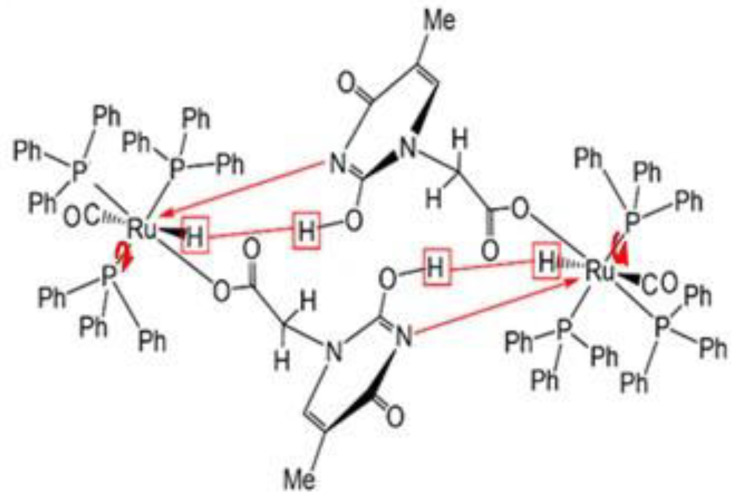
Description of the “synchronic” acid–base reaction of the carboxyl units and the alcohol moieties to the ruthenium hydrides, driven by the release of H_2_ favoring entropy, together with the N-nucleophilic attack by the iminol units, which induce the cleavage of the opposite phosphine ligand to achieve the observed stereochemistry of the final macrocycle of **4**.

As an alternative description of a further plausible mechanism, inspired by the stereogeometry of the final product (see Figure 8), we report the following nucleophilic reciprocal attack (see Figure 12).

## 3. Materials and Methods

### 3.1. General

All reactions were routinely carried out under an argon atmosphere, using the standard Schlenk techniques. The solvents were distilled immediately before use under nitrogen from the appropriate drying agents. Chromatographic separation was carried out on columns of dried celite. The glassware was oven-dried before use. The infrared spectra were recorded at 298 K on a PerkinElmer Spectrum 2000 FT-IR (Fourier transform infrared) spectrophotometer (Waltham, MA, USA), the electrospray ionization mass spectrometry (ESI MS) spectra were recorded on a Waters Micromass ZQ 4000 (Milford, MA, USA), with the samples dissolved in CH_3_OH. All the deuterated solvents were degassed before use. All NMR measurements were performed on a Mercury Plus 400 instrument (Oxford Instruments, Abingdon-on-Thames, UK). The chemical shifts for ^1^H and ^13^C were referenced to internal TMS. All NMR spectra were recorded at 298 K. The decomposition point (dec. point) was measured on a Büchi 535 apparatus (Flawil, Switzerland). All the experimental and calculated yields were referenced to the precursor **1**. All the reagents were commercial products (Aldrich, Saint Louis, MI, USA) of the highest purity available and were used as received. The (RuCl_3_·xH_2_O) was purchased from Strem (Bischheim, France) and used as received. The compound (Ru(H)_2_(CO)(PPh_3_)_3_) (**1**) was prepared by published methods [17].

### 3.2. Experimental Procedure

#### 3.2.1. Synthesis of the Complex k^1^(O), k^2^(O,O)-(Ru(CO)(PPh_3_)_2_THAc) (**5**)

A refluxing toluene solution of (Ru(H)_2_(CO)(PPh_3_)_3_) (100 mg, 0.109 mmol) were added under stirring to two equivalents of thymine acetic acid (40 mg, 0.218 mmol). After 4 h, the absorption of Ru-CO at 1940 cm^−1^ disappeared. After drying under a vacuum, the light brown solid was washed with Et2O (3 × 10 mL) to remove the released PPh_3_. The crystallization that occurred in the CDCl_3_ produced white crystals of **5** (0.095 mmol, 97 mg, 87%). The same reaction was carried out by adding an excess of THAcH or two equivalents consecutively until the infrared (IR) absorption bands of the starting material disappeared.

The spectroscopic characterization, including the mass spectra of **5**, has already been reported in previous studies [1].

#### 3.2.2. Synthesis of the Complexes k^1^(O), k^2^(N,O)-(Ru(CO)(PPh_3_)_2_THAc)_2_ (**4**) + k^1^(O), k^2^(O,O)-Ru(CO)(PPh_3_)_2_(THAc)_2_) (**5**)

The ligand thymine acetic acid (20 mg, 0.109 mmol) and (Ru(H)_2_(CO)(PPh_3_)_3_) (100 mg, 0.109 mmol) were dissolved in 10 mL of toluene and refluxed until the initial Ru-CO IR absorption of **1** at 1941 cm^−1^ disappeared. After 3 h of refluxing in toluene, the mixture was cooled to room temperature and the solvent was evaporated under a vacuum. Then the solid was dissolved in dichloromethane and filtered on a celite pad. After evaporation of the solvent, the dark brown solid was re-dissolved in CDCl_3_ and cooled to −20 °C. After standing for 30 days at −20 °C, white crystals belonging to the macrocyclic dimer complex (**4**) (30 mg, 0.018 mmol, 33%) and to the doubly coordinated species of **5** (20 mg, 0.020 mmol, 18%) were collected and filtered.

IR (KBr, cm^−1^): ν CH, 3059 m; Ru-CO 1975 vs. ν_asym_ C(O)O 1654 vs. ν_sym_ C(O)O 1624 vs. C=N-C(O) 1535 vs. ν C=C 1473 vs. 1437 s; ν C-O 1340 s. ^1^H NMR (300 MHz, 25 °C CDCl_3_) δ (ppm): 7.74–7.29 (PPh_3_, 30H) 5.75 (s, 1H), 3.17 (s, 2H), 1.91 (s, 3H). ^13^C NMR (101 MHz, CDCl_3_) δ (ppm): 191.97 (C=O THAc), 167.91 (C(O)O THAc), 135–127 (PPh_3_), 125.17 (CH), 68.30 (N-CH_2_), 38.88 (CH_3_). ^31^P NMR (162 MHz, CDCl_3_) δ (ppm): 46.51 (d, ^2^*J_PP_* = 25.7 Hz), 43.09 (d, ^2^*J_PP_* = 25.7 Hz). Dec. point 185–187 °C. Elemental analysis: calculated C, 63.15; H, 4.46; O, 9.56; P, 7.40; actual C, 61.83; H, 4.57; O, 9.07; P, 7.31.

### 3.3. X-ray Crystallography

The X-ray intensity data were measured on a Bruker Apex II CCD diffractometer. The cells’ dimensions and the orientation matrix were initially determined from a least-squares refinement on the reflections measured in three sets of 20 exposures, collected in three different ω regions, and eventually refined against all data. A full sphere of the reciprocal space was scanned in steps of 0.3° ω. The software SMART (version 5.051) was used for collecting the frames of data, indexing the reflections, and determining the lattice parameters. The collected frames were then processed for integration by the SAINT program, and an empirical absorption correction was applied by using SADABS. The structure was solved by direct methods (SIR 2004) and subsequent Fourier syntheses, and refined by the full-matrix least-squares on F2 (SHELXTL) [49,50,51,52] using anisotropic thermal parameters for all non-hydrogen atoms. The asymmetric unit contained one CHCl_3_ molecule and one H_2_O molecule.

CCDC-2,252,045 contains the supplemental crystallographic data from this study. The data can be obtained free of charge at www.ccdc.cam.ac.uk/conts/retrieving.html, (accessed on 27 march 2023), or by contacting The Cambridge Crystallographic Data Centre CB2 1EZ, UK; fax: +44 1223 33603, e-mail: deposit@ccdc.cam.ac.uk).

### 3.4. Computational Details

All DFT calculations were performed using the ORCA 5.0.3 suite of quantum chemistry programs [53]. The geometries were optimized in a vacuum using the M06L function [54] and the def2-TZVP basis [55]. Dispersion corrections were also accounted for by following the DFT-D3 procedure (with zero damping functions), as suggested by Grimme et al. [56]. The vibrational frequencies were calculated at the optimized geometries to check the stability of the stationary points. The free energies at the boiling temperature of toluene (383.8K) were evaluated by applying a scale factor of 0.9824 to the vibrational frequencies, which was adequate for the present combination of functional DFT and the basis set [57] and a hypothetical pressure of 294.7 atm for the toluene solvent [58], to correct the overestimation of the entropic contributions. The final single-point energy calculations at the previously optimized geometries were performed with the large def2-QZVPP basis [55] and the M06 function [59], with the inclusion of the effects of solvation (with a toluene solvent) through the SMD model [60] and the interactions of dispersion [56]. The final energy of each structure, which was used to evaluate the relative free energies of the various products and intermediates, was determined by summing the difference between the def2-TZVP electronic energy and the free energy to the single-point electronic energy of def2-QZVPP.

## 4. Conclusions

By reacting **1** with one equivalent of THAcH after standing for 30 days at −20 °C in CDCl_3_, both **4** and **5** were formed. This unexpected result can be explained by postulating the intercepted intermediates as key reaction steps. The self-aggregation of the rapidly formed monohapto-acetate **2a,** followed by the mutual addition of heteroleptic (N,O) produced **4**. Conversely, the homoleptic chelation of **2b** to form the chelated species **3** was kinetically competitive, promoting the further incorporation of the THAc ligand to produce **5**.

The energetic considerations calculated by DFT enabled us to interpret the mechanism through the penta-coordinated derivatives, postulated as rather low energetic transition states. The *trans–cis* rearrangement required the occurrence of a much higher energy barrier than the nucleophilic attack of the *mer*-monohapto acetate of **2** on the reciprocal Ru site, which released an apical ligand, to produce the resulting *cis-_P,P_* stereochemistry. The proposed mechanism supports the facile promotion of H to explain the tautomerization from keto-amino to iminol species, exhibiting a stronger Ru-nucleophilic reaction of the k^1^(O)-coordinated thymine ring by regulating the distribution of the kinetic products. The observed dynamic behavior of the particular aptitude for the transfer of H, by keeping the metallic scaffold, might suggest further tailored catalytic processes, such as isomerization, as in the case of the chelate homoleptic k^2^(O,O)- species [13] or novel insight into therapeutic antitumor agents for the versatile heteroleptic k^2^(N,O) compounds [3]. Trapping small molecules such as solvents or CO_2_, or sequestering alkaline metal ions may also constitute some visionary applications of the macromolecule **4**, considering its behavior as an organometallic crown ether. These targeted topics could be the subjects of novel perspectives and future investigations.

## Figures and Tables

**Table 1:** Selected bond lengths (Å) and angles (°) of **4** compared with the free THAcH. Adapted with permission from Ref. [18]. 2004, Liu M.C.

**Thymine Acetate**	**4**	**THAcH**
C6-O4	1.23 (1)	1.241 (2)
C6-N2	1.35 (1)	1.371 (2)
C7-N2	1.341 (9)	1.379 (2)
C7-O3	1.280 (8)	1.213 (2)
C7-N1	1.358 (9)	1.376 (2)
C3-C4	1.33 (1)	1.338 (2)
C3-N1	1.38 (1)	1.375 (2)
**Metal Center**		
Ru-P1	2.355 (2)	
Ru-P2	2.348 (2)	
Ru-O1	2.108 (5)	
Ru-O3	2.216 (5)	
Ru-N2	2.109 (7)	
Ru-C8	1.81 (1)	

## Data Availability

Not applicable.

## Supplementary Materials

The following supporting information can be downloaded at https://www.mdpi.com/article/10.3390/molecules28103983/s1. NMR spectra (Figures S1–S3 in Supplementary File). IR spectra (Figures S4–S6 in Supplementary File). Mass spectra (Figures S7–S10 in Supplementary File). Computations (Figures S11–S13 in Supplementary File). Crystal structure (Table S1, Figure S14 in Supplementary File). PXRD (Figure S15 in Supplementary File).
