# Supplementary material for: Ru-Controlled Thymine Tautomerization Frozen by a k1(O)-, k2(N,O)-Metallacycle: An Experimental and Theoretical Approach
Source: Molecules. 2023 May 9;28(10):3983. doi: 10.3390/molecules28103983 (PMC10223689; doi:10.3390/molecules28103983)
Supplement: Supplementary file 1 [file molecules-28-03983-s001.zip › molecules-2349016-supplementary.pdf]

Table of Contents

NMR SPECTRA 2
- S1 2
- S2 3
- S3 4

IR SPECTRA 5
- S4 5
- S5 5
- S6 6

MASS SPECTRA 6
- S7 6
- S8 7
- S9 8
- S10 9

COMPUTATIONAL 10
- S11 10
- S12 11

S13 12

CRYSTAL STRUCTURE 13
- Table S1 13
- S14 14

PXRD 14
- S15 14

NMR SPECTRA

S1

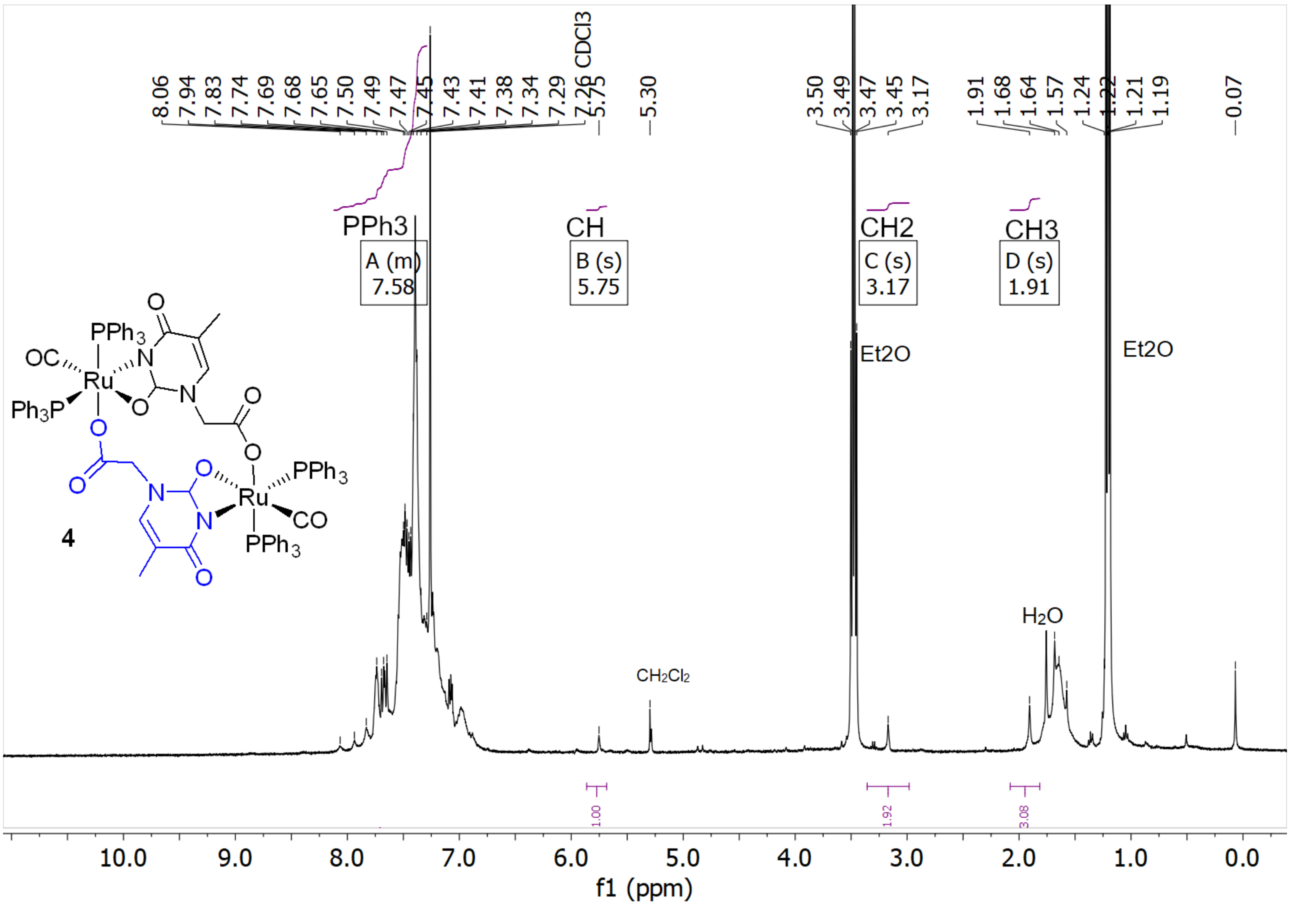

Figure S1: *^{1}H NMR spectrum of* ***4*** in $CDCl_3$ (300 Mhz)

S2

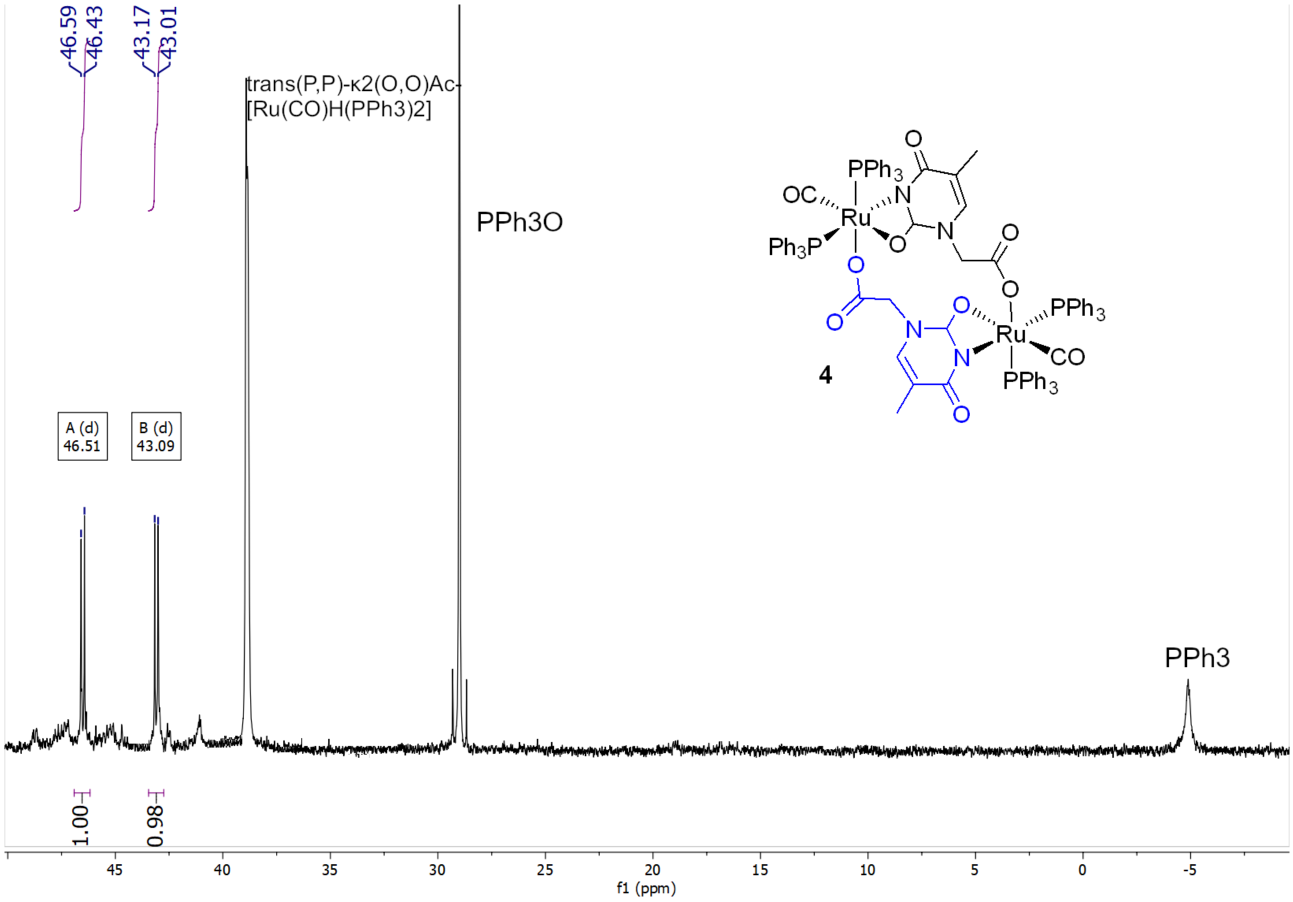

Figure S2: *^{31}P NMR spectrum of **4** in $CDCl_3$ (162 Mhz)*

S3

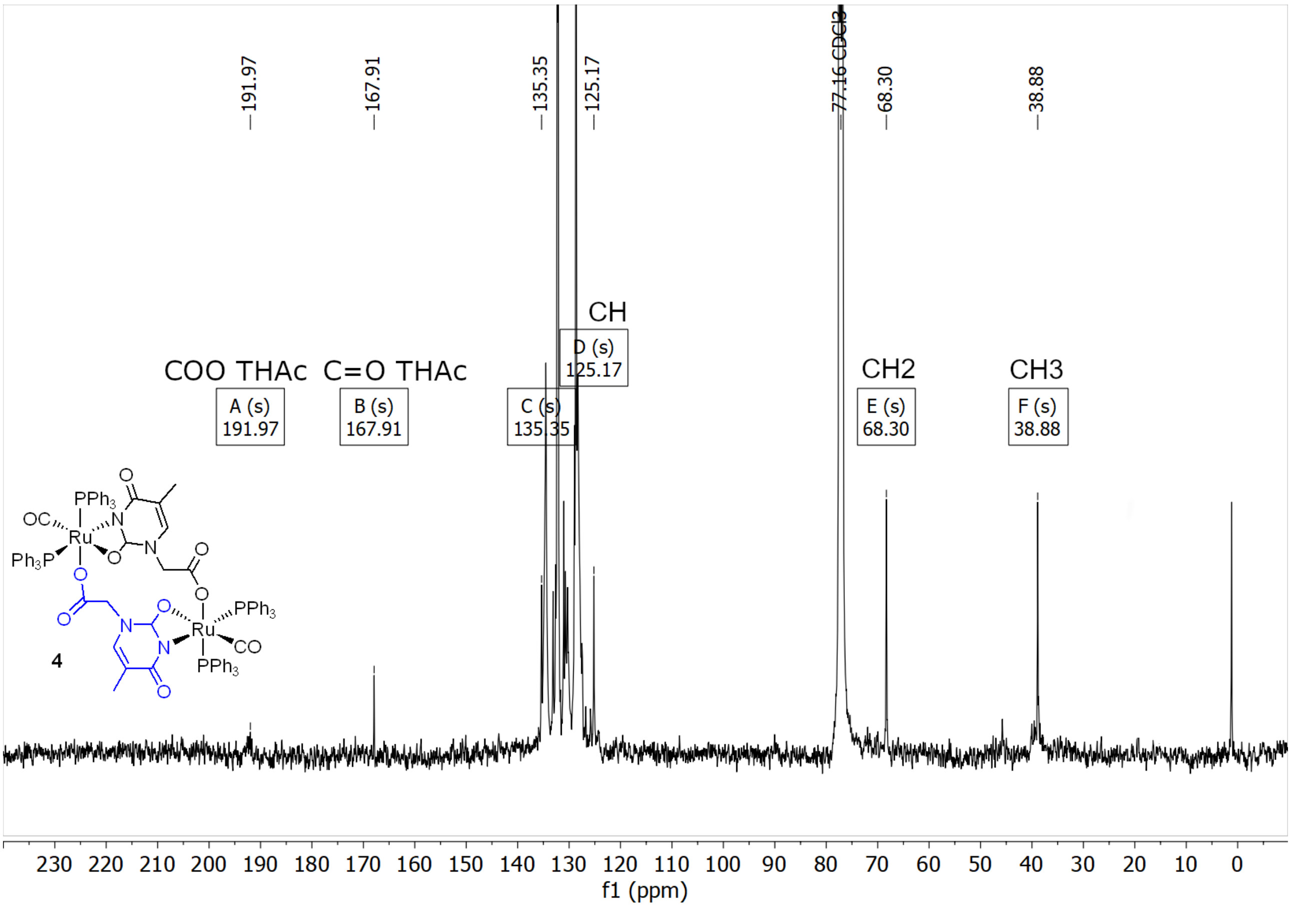

Figure S3: *^{13}C NMR spectrum of **4** in $CDCl_3$ (101 Mhz)*

IR SPECTRA

S4

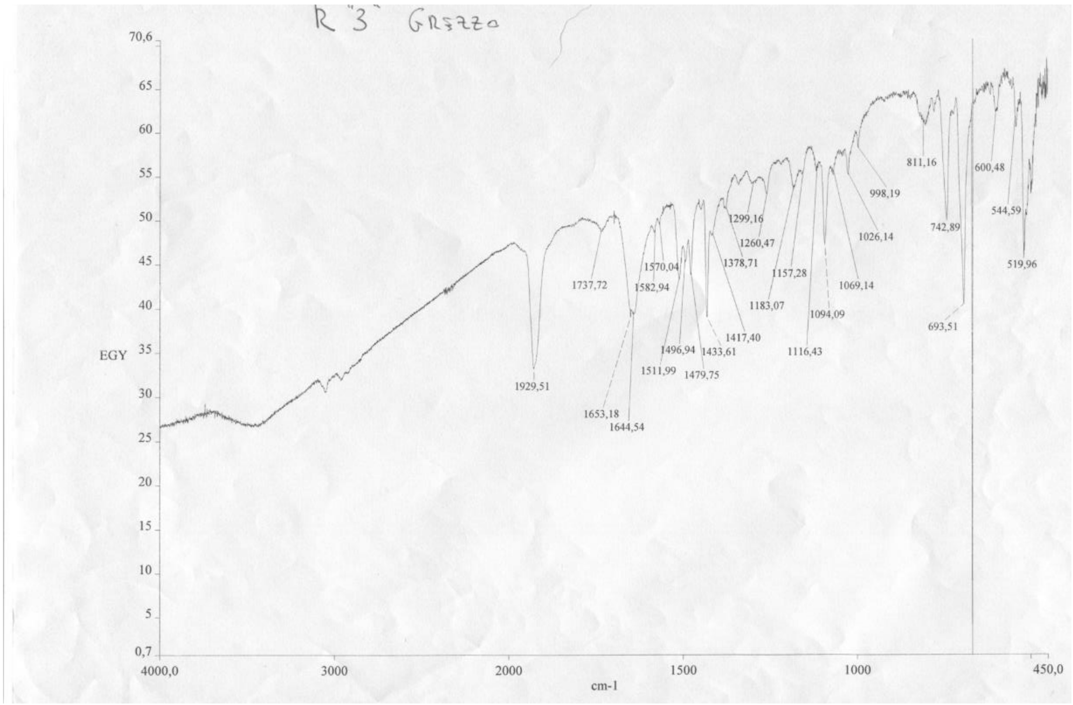

Figure **S4:** IR spectrum of k 1 (O)-**2a** enolic form. The band at 1511 cm-1 is attributable to the incipient transformation to the enol moiety of the precursor of **4.**

S5

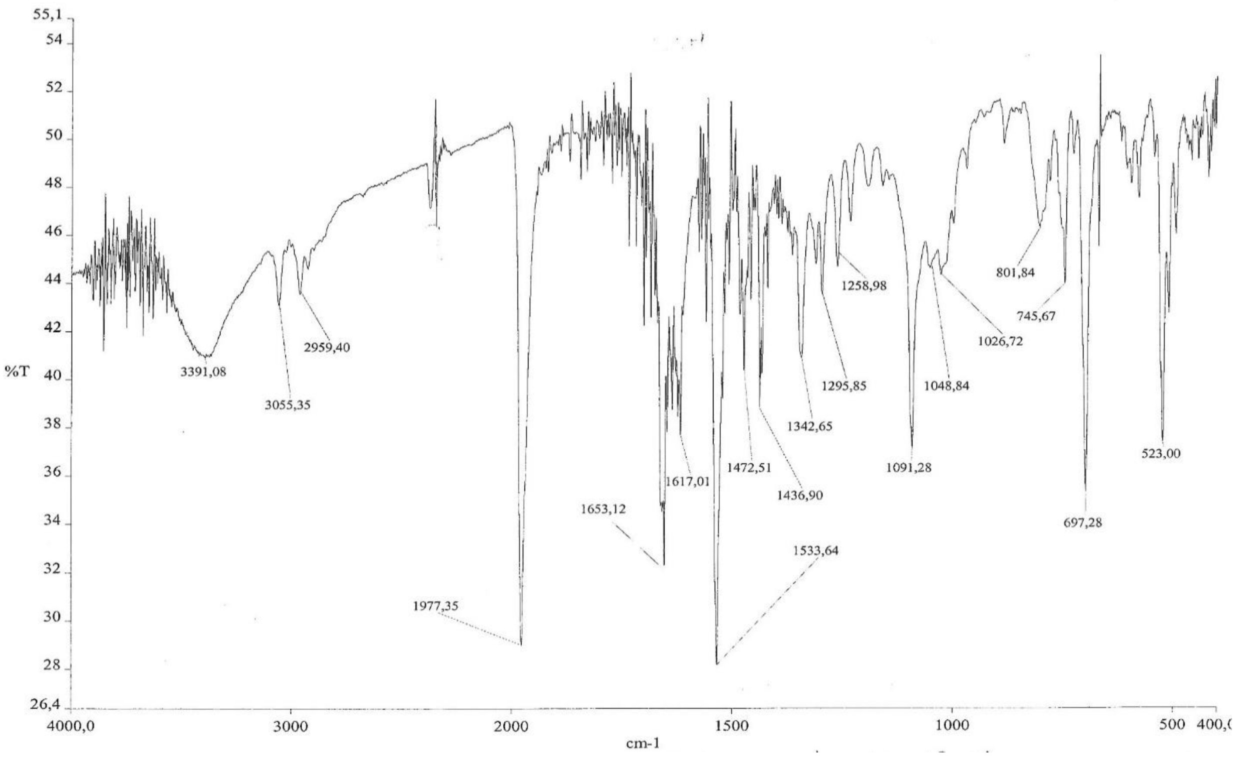

Figure S5: IR spectrum of microcrystalline powder **4** of a different preparation: the band atν = 1533.64 is characteristic for the lactim tautomeric form.

S6

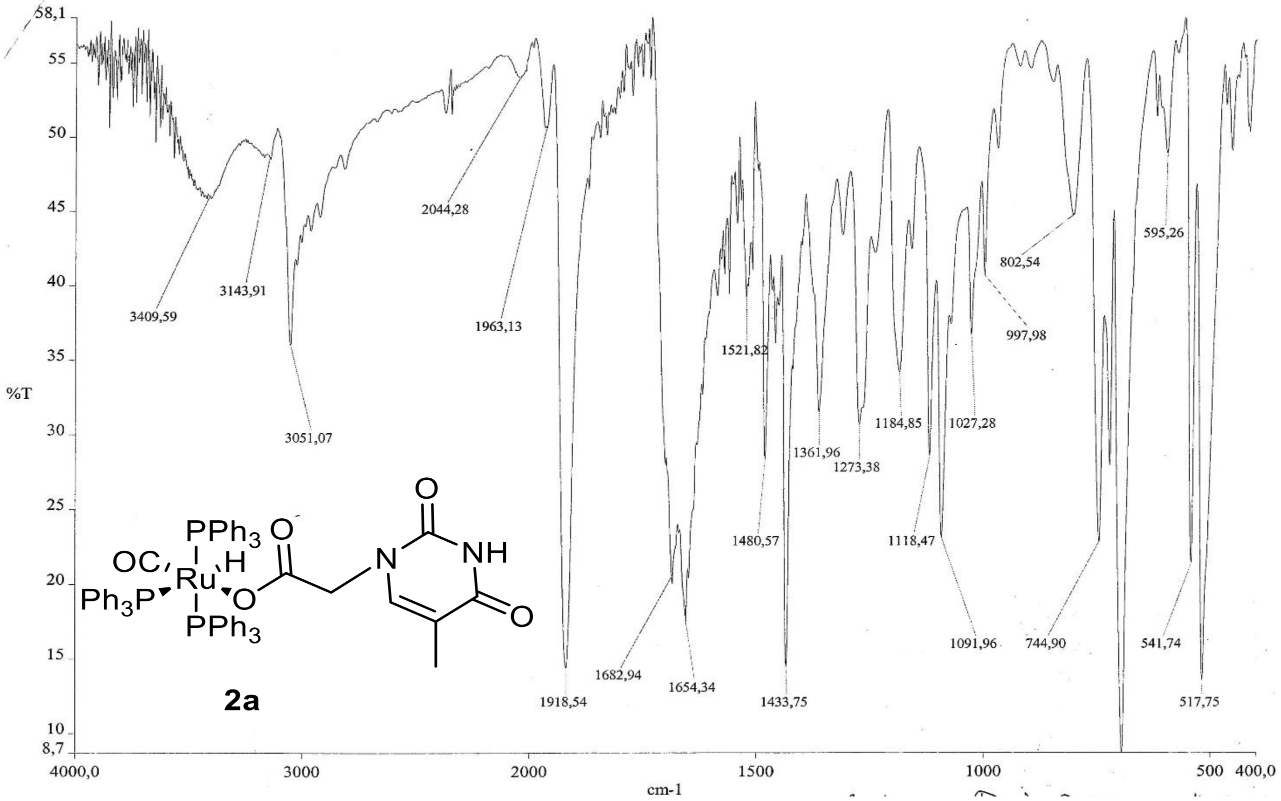

Figure S6: IR spectrum of KETO **2a**, after extraction in DCM/Et2 1:1

MASS SPECTRA

S7

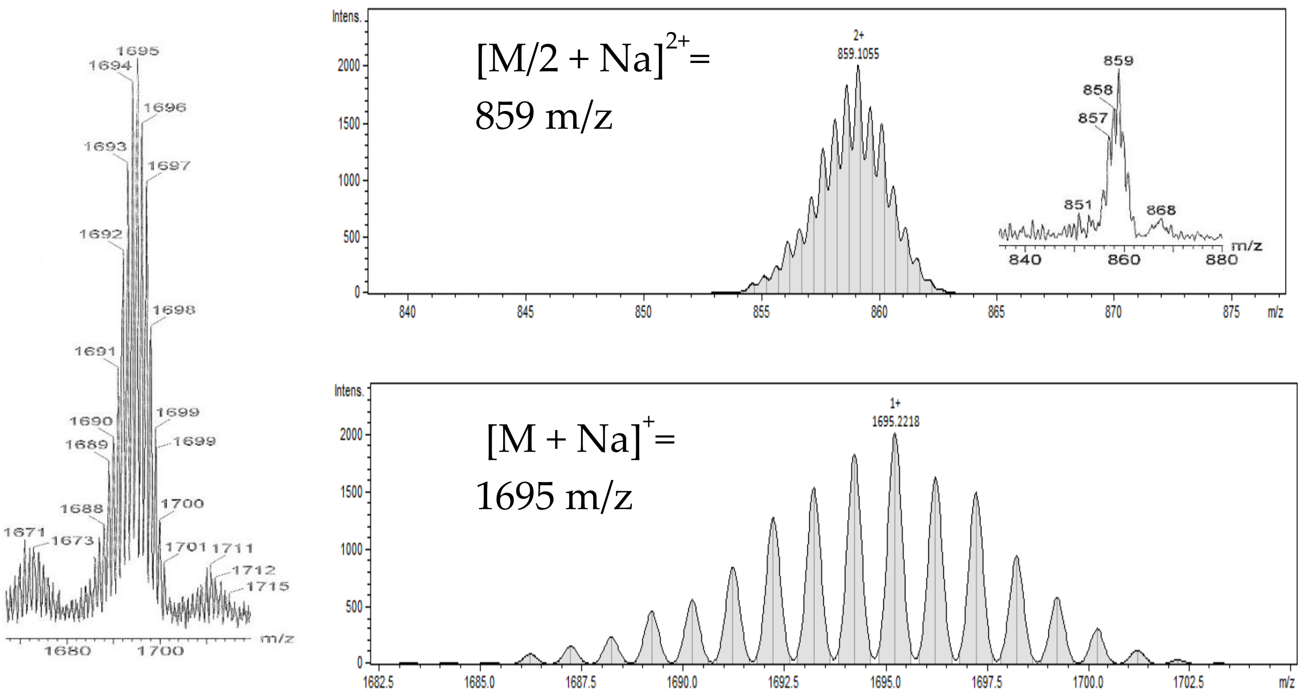

Figure S7: Mass spectrum of **4** compared to simulations.

A

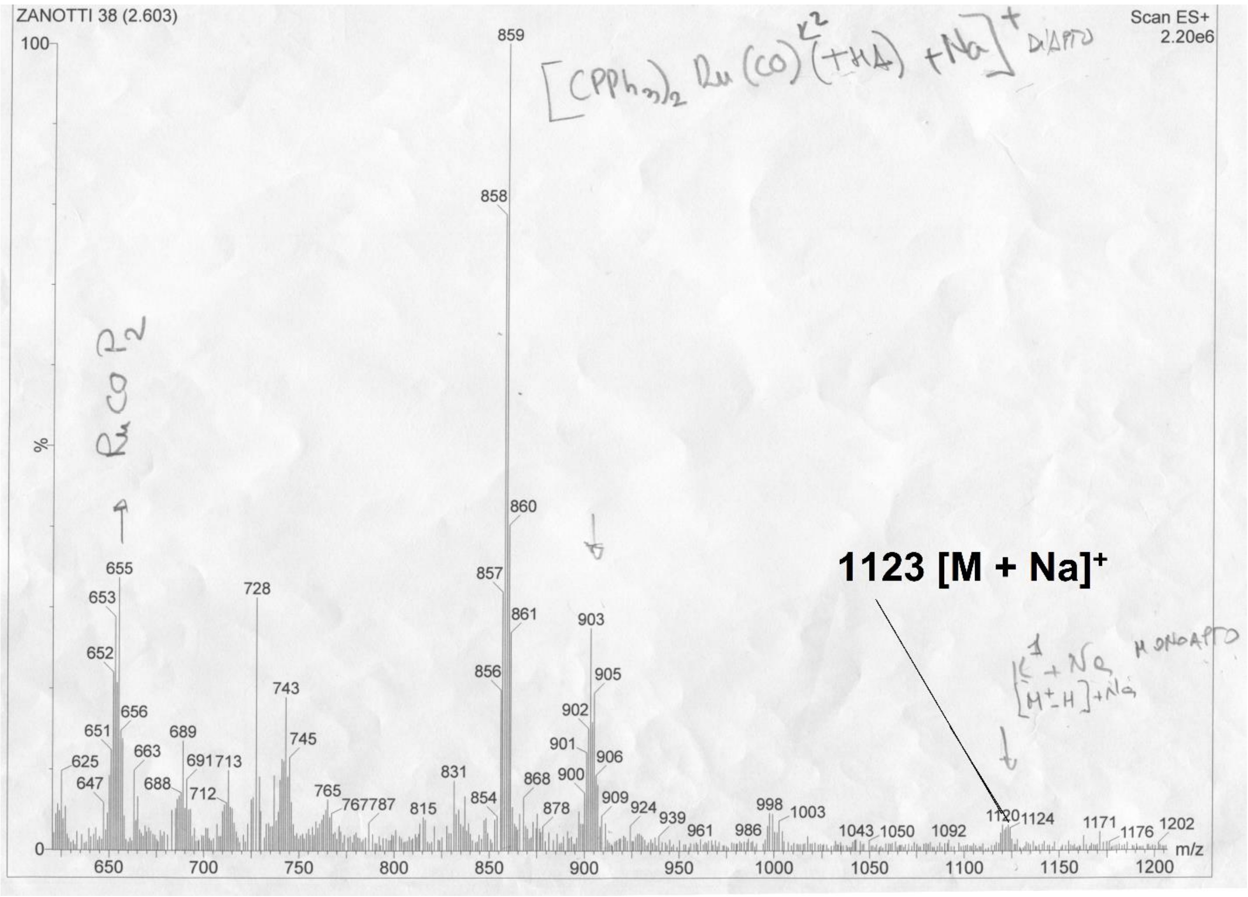

B

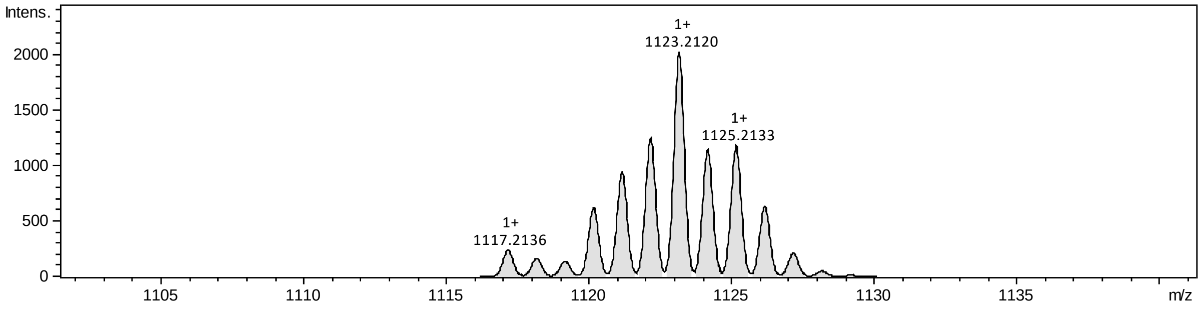

Figure S8: *(**A**) Mass spectrum of* k^1*(O)-**2a**.* (***B***) simulation of [M + Na]$^+$

S9

A

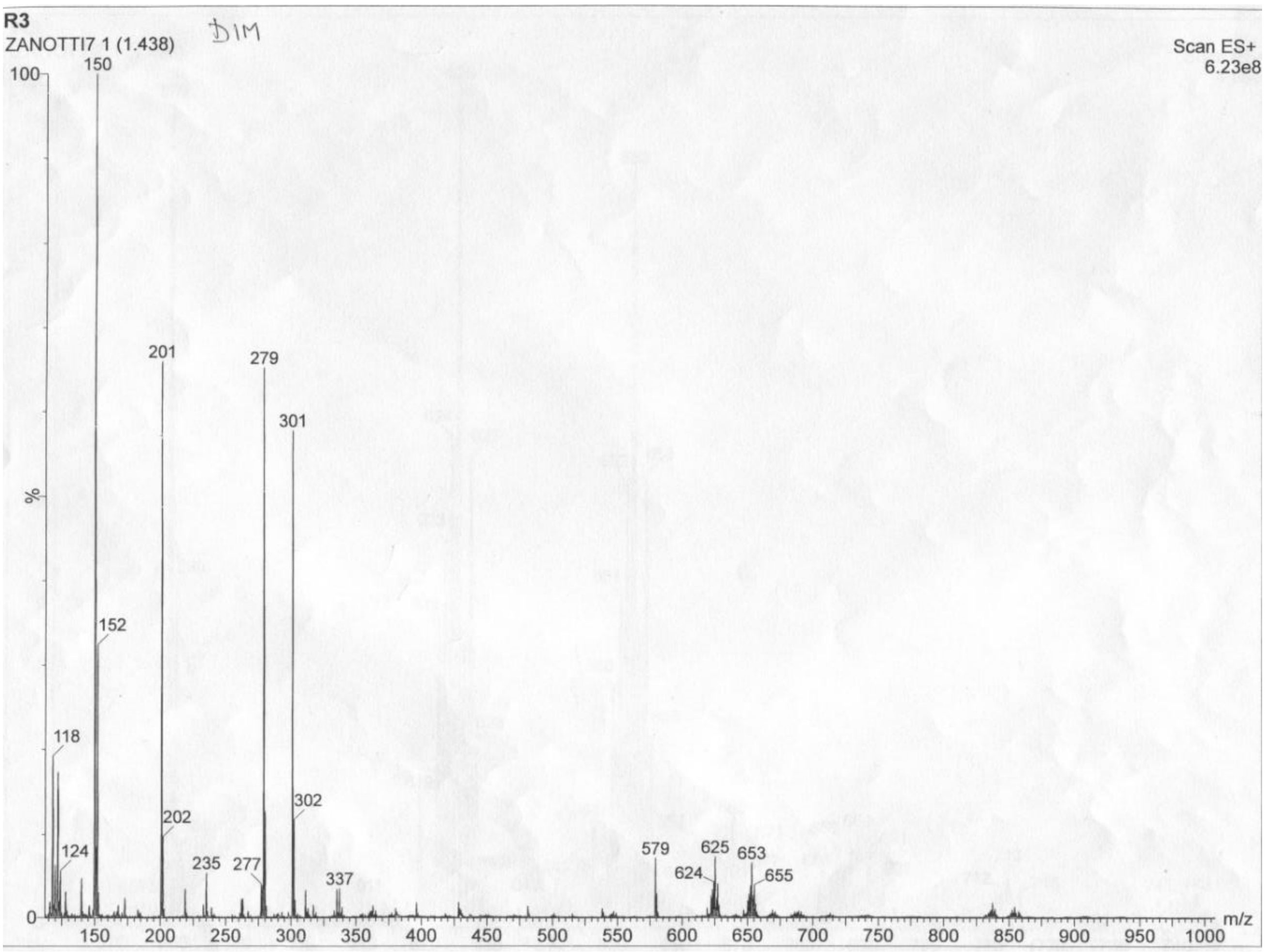
R3
ZANOTTI7 1 (1.438)
DIM
Scan ES+
6.23e8
100
%
0
150
201
279
301
152
118
302
202
124
235
277
337
579
625
624
653
655
m/z
150
200
250
300
350
400
450
500
550
600
650
700
750
800
850
900
950
1000

B

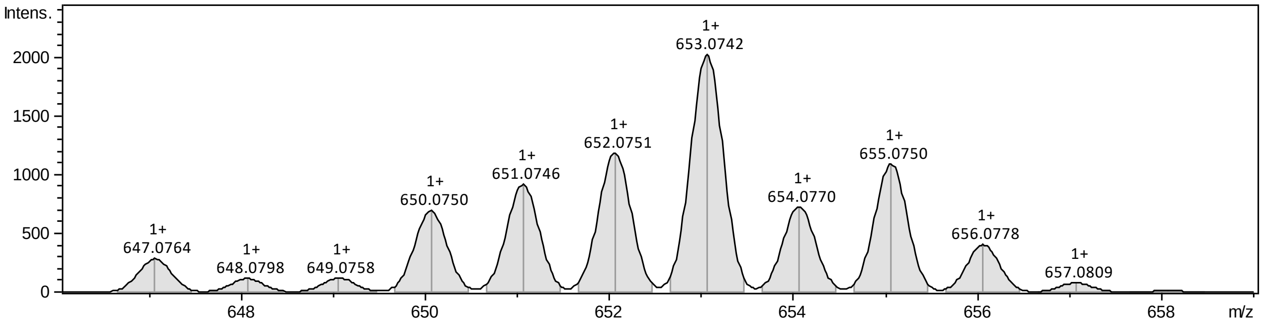
Intens.
2000
1500
1000
500
0
1+
653.0742
1+
652.0751
1+
655.0750
1+
651.0746
1+
650.0750
1+
654.0770
1+
656.0778
1+
647.0764
1+
648.0798
1+
649.0758
1+
657.0809
648
650
652
654
656
658
m/z

Figure S9*: **(A)** Mass spectrum of k²(O,O)-**3*** (positive mode). *(**B**)* simulation of [M – THAc]+

S10

A

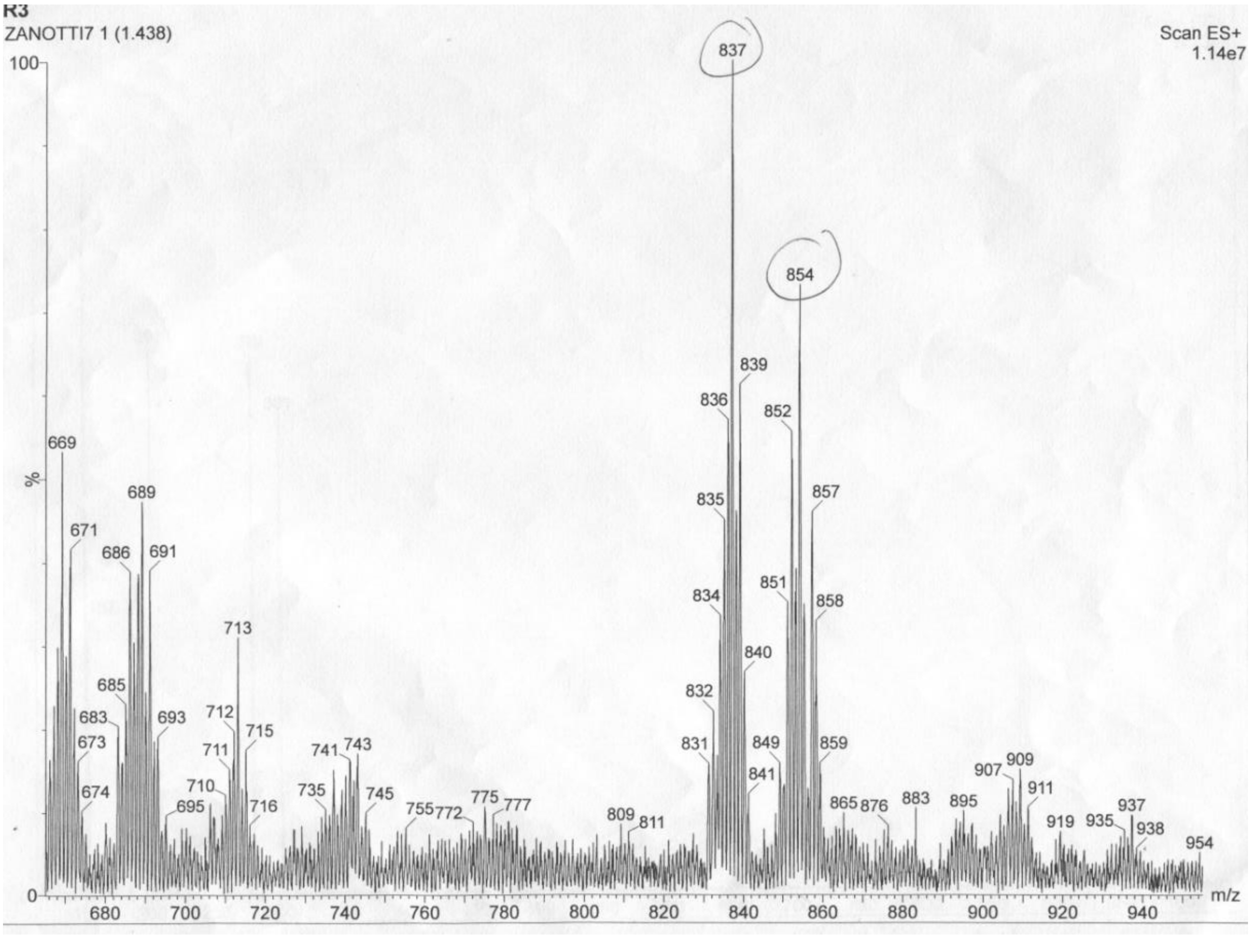

B

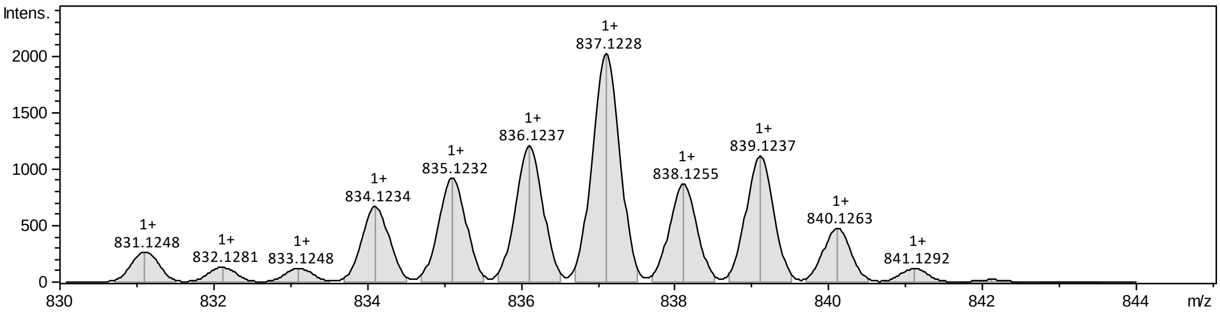

Figure S10*: (**A**) Mass spectrum of k²(O,O)-**3*** (positive mode, m/z: 680 – 940). (**B**) Simulation of [M – H]⁺

COMPUTATIONAL

S11

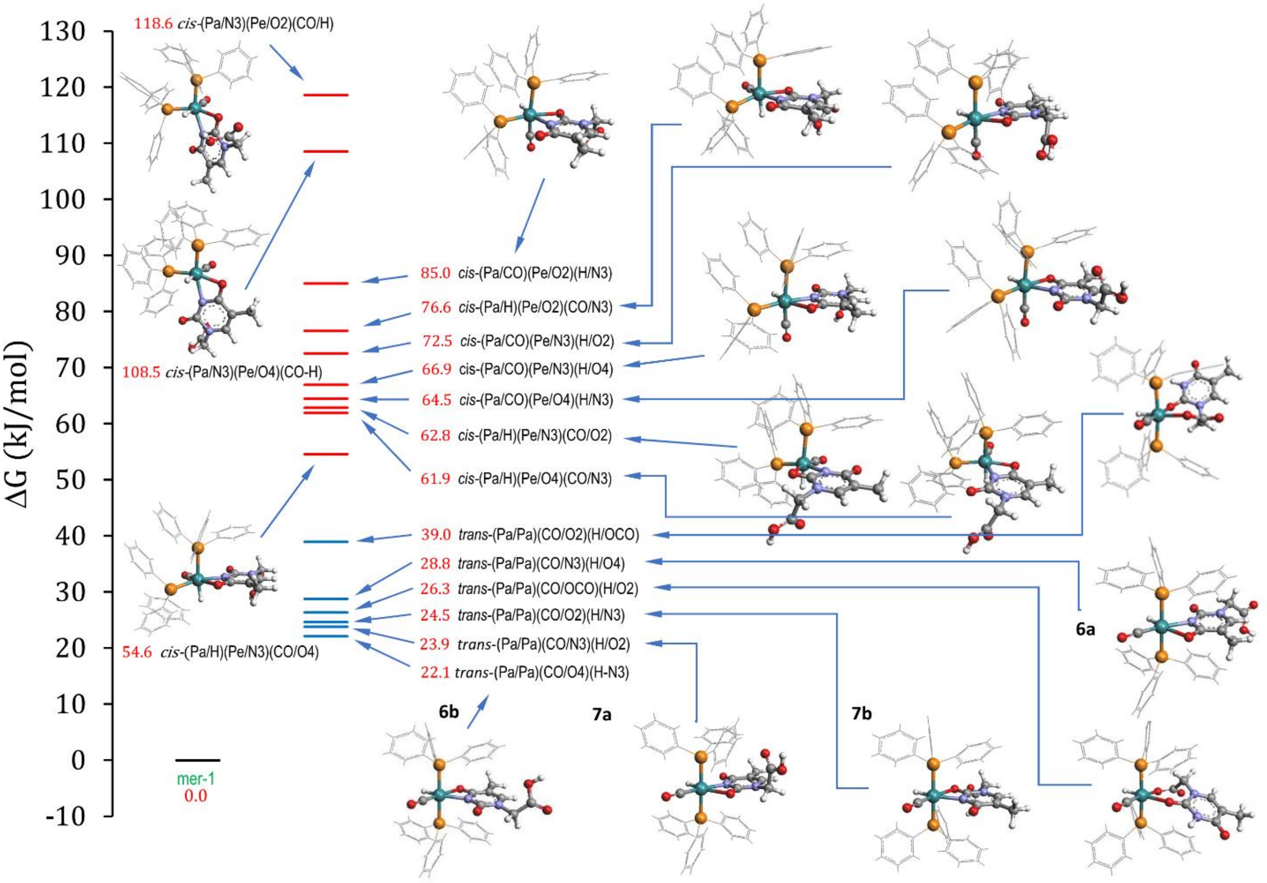

Figure S11: DFT calculations of all four membered- k2(N,O)- heteroleptic and k2(O,O)- heptacycles. All energies are calculated relative to the energy of mer-1 + thymine-acetic acid reactants. All species are named using the following scheme: the three couples of ligands at opposite vertex of the octahedron are enclosed in parentheses, with the additional specification of relative phosphine position. Legend: Pa=axial phosphine, Pe=equatorial phosphine, CO=carbonyl, H=hydride, OCO=thymine carboxylate, O2=thymine oxygen in 2 position, N3=thymine nitrogen in 3 position, O4=thymine oxygen in 4 position

S12

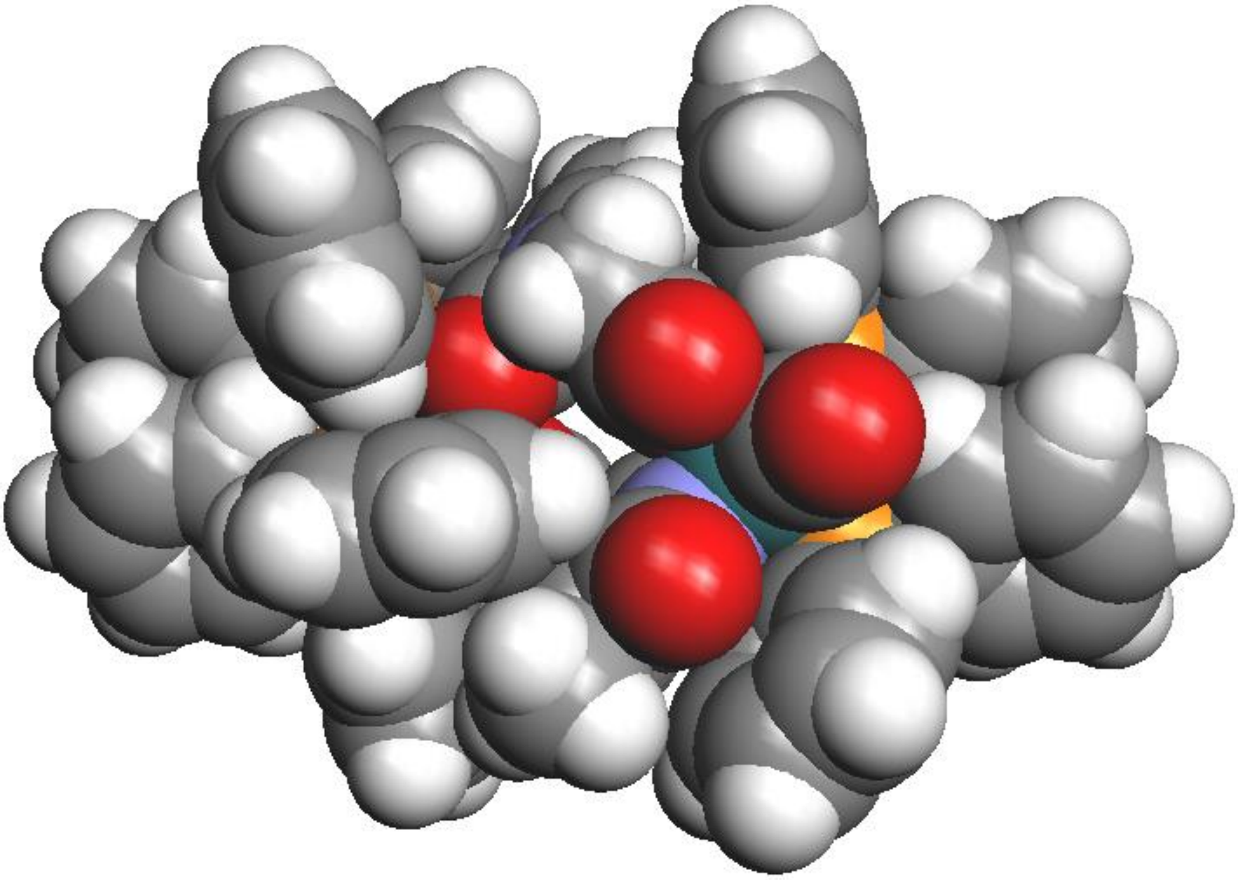

Figure S12: Space filling of **4**

S13

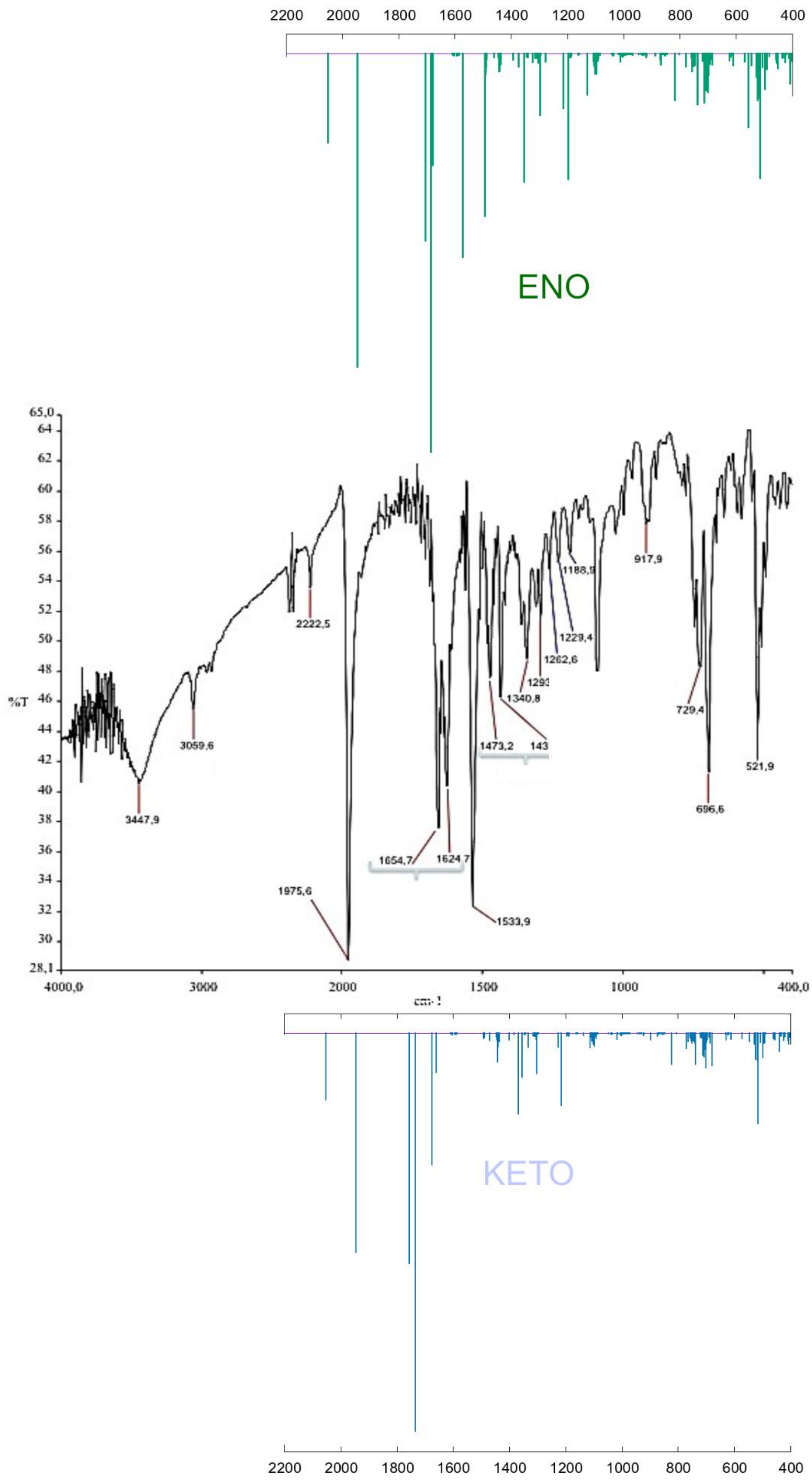

Figure S13: Comparison between the experimental IR spectrum of DFT-simulated IR spectra of keto and Enol form relative to the dimer **4**.

CRYSTAL STRUCTURE

Table S1

Table S1. Crystal data and experimental details for **4**

Compound	**4**
Formula	$C_{88}H_{72}N_4O_{10}P_4Ru_2.2CHCl_3.2H_2O$
Fw	1942.25
T, K	296(2)
λ, Å	0.71073
Crystal symmetry	Monoclinic
Space group	$P2_1/c$
a, Å	10.220(2)
b, Å	29.771(7)
c, Å	14.713(3)
α	90
β	90.005(2)
γ	90
Cell volume, $Å^3$	4476.7(17)
Z	2
D_c, Mg m^{-3}	1.441
$\mu(Mo–K_\alpha)$, mm^{-1}	0.649
F(000)	1976
Crystal size/ mm	0.15 x 0.05 x 0.05
θ limits, °	1.368 to 25.000
Reflections collected	36382
Unique obs. Reflections $[F_o > 4\sigma(F_o)]$	7816 [R(int) = 0.1264]
Goodness-of-fit-on F^2	0.983
R_1 (F)[a], wR_2 (F^2) [I > 2σ(I)][b]	0.0738, 0.1418
Largest diff. peak and hole, e. $Å^{-3}$	0.773 and -0.616

[a] $R_1 = \Sigma||F_o|-|F_c||/\Sigma|F_o|$. [b] $wR_2 = [\Sigma w(F_o^2-F_c^2)^2/\Sigma w(F_o^2)^2]^{1/2}$ where $w = 1/[\sigma^2(F_o^2) + (aP)^2 + bP]$ where $P = (F_o^2 + F_c^2)/3$.

S14

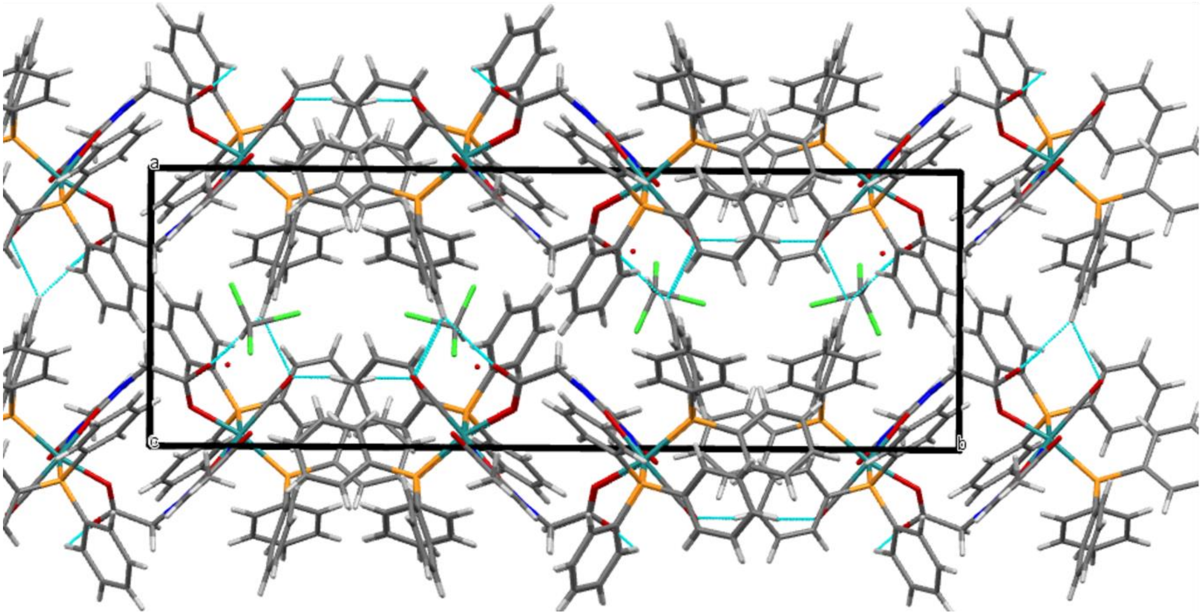

Figure S14: View down the c axis of the crystal packing of **4.** Light blue dotted lines represent H bonding interactions**.**

PXRD

S15

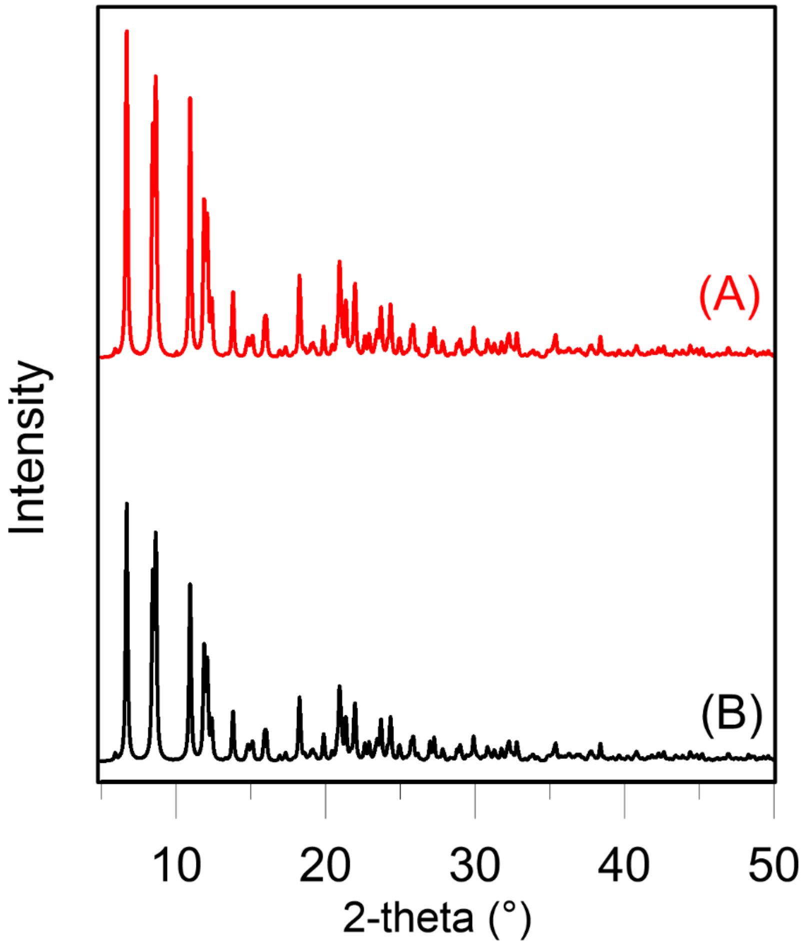

Figure S15: Comparison between the experimental (**A**) and simulated (**B**) PXRD spectra of **4**, which supports the phase purity of compound **4**.
